# Alterations in Abundance and Compartmentalization of miRNAs in Blood Plasma Extracellular Vesicles and Extracellular Condensates during HIV/SIV Infection and Its Modulation by Antiretroviral Therapy (ART) and Delta-9-Tetrahydrocannabinol (Δ^9^-THC)

**DOI:** 10.3390/v15030623

**Published:** 2023-02-24

**Authors:** Steven Kopcho, Marina McDew-White, Wasifa Naushad, Mahesh Mohan, Chioma M. Okeoma

**Affiliations:** 1 Department of Pharmacology, Stony Brook University Renaissance School of Medicine, Stony Brook, NY 11794-8651, USA; 2 Host Pathogen Interaction Program, Southwest National Primate Research Center, Texas Biomedical Research Institute, San Antonio, TX 78227-5302, USA; 3 Department of Pathology, Microbiology, and Immunology, New York Medical College, Valhalla, NY 10595-1524, USA; 4 Lovelace Biomedical Institute, Albuquerque, NM 87108-5127, USA

**Keywords:** extracellular vesicles (EVs), extracellular condensates (ECs), miRNA, SIV, THC, cART

## Abstract

In this follow-up study, we investigated the abundance and compartmentalization of blood plasma extracellular miRNA (exmiRNA) into lipid-based carriers—blood plasma extracellular vesicles (EVs) and non-lipid-based carriers—extracellular condensates (ECs) during SIV infection. We also assessed how combination antiretroviral therapy (cART), administered in conjunction with phytocannabinoid delta-9-tetrahydrocannabinol (THC), altered the abundance and compartmentalization of exmiRNAs in the EVs and ECs of SIV-infected rhesus macaques (RMs). Unlike cellular miRNAs, exmiRNAs in blood plasma may serve as minimally invasive disease indicators because they are readily detected in stable forms. The stability of exmiRNAs in cell culture fluids and body fluids (urine, saliva, tears, cerebrospinal fluid (CSF), semen, blood) is based on their association with different carriers (lipoproteins, EVs, and ECs) that protect them from the activities of endogenous RNases. Here, we showed that in the blood plasma of uninfected control RMs, significantly less exmiRNAs were associated with EVs compared to the level (30% higher) associated with ECs, and that SIV infection altered the profile of EVs and ECs miRNAome (Manuscript 1). In people living with HIV (PLWH), host-encoded miRNAs regulate both host and viral gene expression, which may serve as indicators of disease or treatment biomarkers. The profile of miRNAs in blood plasma of PLWH (elite controllers versus viremic patients) are different, indicating that HIV may alter host miRNAome. However, there are no studies assessing the effect of cART or other substances used by PLWH, such as THC, on the abundance of exmiRNA and their association with EVs and ECs. Moreover, longitudinal exmiRNA profiles following SIV infection, treatment with THC, cART, or THC+cART remains unclear. Here, we serially analyzed miRNAs associated with blood plasma derived EVs and ECs. **Methods:** Paired EVs and ECs were separated from EDTA blood plasma of male Indian rhesus macaques (RMs) in five treatment groups, including VEH/SIV, VEH/SIV/cART, THC/SIV, THC/SIV/cART, or THC alone. Separation of EVs and ECs was achieved with the unparalleled nano-particle purification tool ─PPLC, a state-of-the-art, innovative technology equipped with gradient agarose bead sizes and a fast fraction collector that allows high resolution separation and retrieval of preparative quantities of sub-populations of extracellular structures. Global miRNA profiles of the paired EVs and ECs were determined with RealSeq Biosciences (Santa Cruz, CA) custom sequencing platform by conducting small RNA (sRNA)-seq. The sRNA-seq data were analyzed using various bioinformatic tools. Validation of key exmiRNA was performed using specific TaqMan microRNA stem-loop RT-qPCR assays. **Results:** We investigated the effect of cART, THC, or both cART and THC together on the abundance and compartmentalization of blood plasma exmiRNA in EVs and ECs in SIV-infected RMs. As shown in Manuscript 1 of this series, were in uninfected RMs, ~30% of exmiRNAs were associated with ECs, we confirmed in this follow up manuscript that exmiRNAs were present in both lipid-based carriers—EVs and non-lipid-based carriers—ECs, with 29.5 to 35.6% and 64.2 to 70.5 % being associated with EVs and ECs, respectively. Remarkably, the different treatments (cART, THC) have distinct effects on the enrichment and compartmentalization pattern of exmiRNAs. In the VEH/SIV/cART group, 12 EV-associated and 15 EC-associated miRNAs were significantly downregulated. EV-associated miR-206, a muscle-specific miRNA that is present in blood, was higher in the VEH/SIV/ART compared to the VEH/SIV group. ExmiR-139-5p that was implicated in endocrine resistance, focal adhesion, lipid and atherosclerosis, apoptosis, and breast cancer by miRNA-target enrichment analysis was significantly lower in VEH/SIV/cART compared to VEH/SIV, irrespective of the compartment. With respect to THC treatment, 5 EV-associated and 21 EC-associated miRNAs were significantly lower in the VEH/THC/SIV. EV-associated miR-99a-5p was higher in VEH/THC/SIV compared to VEH/SIV, while miR-335-5p counts were significantly lower in both EVs and ECs of THC/SIV compared to VEH/SIV. EVs from SIV/cART/THC combined treatment group have significant increases in the count of eight (miR-186-5p, miR-382-5p, miR-139-5p and miR-652, miR-10a-5p, miR-657, miR-140-5p, miR-29c-3p) miRNAs, all of which were lower in VEH/SIV/cART group. Analysis of miRNA-target enrichment showed that this set of eight miRNAs were implicated in endocrine resistance, focal adhesions, lipid and atherosclerosis, apoptosis, and breast cancer as well as cocaine and amphetamine addiction. In ECs and EVs, combined THC and cART treatment significantly increased miR-139-5p counts compared to VEH/SIV group. Significant alterations in these host miRNAs in both EVs and ECs in the untreated and treated (cART, THC, or both) RMs indicate the persistence of host responses to infection or treatments, and this is despite cART suppression of viral load and THC suppression of inflammation. To gain further insight into the pattern of miRNA alterations in EVs and ECs and to assess potential cause-and-effect relationships, we performed longitudinal miRNA profile analysis, measured in terms of months (1 and 5) post-infection (MPI). We uncovered miRNA signatures associated with THC or cART treatment of SIV-infected macaques in both EVs and ECs. While the number of miRNAs was significantly higher in ECs relative to EVs for all groups (VEH/SIV, SIV/cART, THC/SIV, THC/SIV/cART, and THC) longitudinally from 1 MPI to 5 MPI, treatment with cART and THC have longitudinal effects on the abundance and compartmentalization pattern of exmiRNAs in the two carriers. As shown in Manuscript 1 where SIV infection led to longitudinal suppression of EV-associated miRNA-128-3p, administration of cART to SIV-infected RMs did not increase miR-128-3p but resulted in longitudinal increases in six EV-associated miRNAs (miR-484, miR-107, miR-206, miR-184, miR-1260b, miR-6132). Furthermore, administration of cART to THC treated SIV-infected RMs resulted in a longitudinal decrease in three EV-associated miRNAs (miR-342-3p, miR-100-5p, miR181b-5p) and a longitudinal increase in three EC-associated miRNAs (miR-676-3p, miR-574-3p, miR-505-5p). The longitudinally altered miRNAs in SIV-infected RMs may indicate disease progression, while in the cART Group and the THC Group, the longitudinally altered miRNAs may serve as biomarkers of response to treatment. **Conclusions:** This paired EVs and ECs miRNAome analyses provided a comprehensive cross-sectional and longitudinal summary of the host exmiRNA responses to SIV infection and the impact of THC, cART, or THC and cART together on the miRNAome during SIV infection. Overall, our data point to previously unrecognized alterations in the exmiRNA profile in blood plasma following SIV infection. Our data also indicate that cART and THC treatment independently and in combination may alter both the abundance and the compartmentalization of several exmiRNA related to various disease and biological processes.

## 1. Introduction

HIV is a disease of great epidemiologic diversity, affecting all countries, ages, sexes, races, genders, and income levels [1,2]. In the absence of treatment, persistent HIV replication results in progressive decline in CD4+ T cell counts and immunodeficiency [3]. Although effective HIV treatment with combination antiretroviral therapy (cART) has prolonged the lives of infected people [4,5], many HIV-associated non-AIDS conditions, including cardiovascular disease, diabetes, renal disease, cancer, gastrointestinal (GI) disorders, and HIV-associated neurocognitive disorder (HAND) occur frequently [6,7,8,9]. Inflammation, chronic immune activation, and immune dysregulation directly and indirectly contribute to these HIV-associated non-AIDS conditions [10]. Hence, controlling inflammation, chronic immune activation, and immune dysregulation may reduce the incidence and severity of HIV-associated non-AIDS conditions despite cART [6,7,8,9].

Cannabis use is common among people living with HIV (PLWH), who often use it to manage disease symptoms [11,12]. Both natural and synthetic forms of Δ^9^ -THC (THC) (available as FDA- approved Marinol^®^ and Syndros^®^), the main psychotropic component of cannabis, have been demonstrated to stimulate appetite and increase body weight, collectively improving the overall well-being of PLWH [13,14]. Interestingly, high-intensity cannabis-smoking HIV-infected individuals have reduced plasma HIV viral load and markers of inflammation [15,16], suggestive of an anti-inflammatory response. THC is a CB1/CB2 receptor partial agonist that exhibits its immunosuppressive and anti-inflammatory effects through the activation of CB2 receptors [17,18]. Additional evidence of the potential anti-inflammatory properties of THC came from an observation that PLWH using marijuana (a component of cannabis) and cocaine showed a reduced and increased immune activation, respectively [19,20], but dual drug (marijuana + cocaine) users showed intermediate levels of immune activation [19]. Controlled studies in SIV-infected RMs indicate that chronic THC treatment slowed disease progression, prolonged survival, conferred protection of the GI tract/mucosa, and attenuated infection-induced inflammation [21,22]. These findings suggest that THC administration may override immune activation and inflammation through their modulation of host factors, such as regulatory RNAs that influence gene expression, such as miRNAs.

MicroRNAs (miRNAs) are abundant within all cell types and are also present in body fluids (such as the blood) in an extracellular form associated with various carriers. The exmiRNA are potential blood-based biomarkers for various diseases. In our initial study (Manuscript 1 of this series), we showed that a significant proportion (approximately 30%) of exmiRNA were associated with ECs compared to EVs. We also showed that SIV infection altered the composition of miRNAs associated with both EVs and ECs, suggesting that the assortment of exmiRNAs into distinct carriers may be a potential new dimension to miRNA-based biomarkers. However, the effects of cART or other substances used by PLWH, such as THC, on exmiRNA abundance and their association with EVs and ECs remains unknown.

In PLWH, miRNAs have been shown to regulate host response to infection. The profile of miRNAs in blood plasma of PLWH (elite controllers versus viremic patients) are different, an indication that HIV may alter the profile of miRNAs [23,24]. However, to our knowledge, no study has assessed the effect of cART or THC on exmiRNA abundance and their association with various extracellular carriers, such as EVs and ECs. These extracellular carriers not only provide protection to miRNAs against RNases but may also facilitate delivery of exmiRNA to distal and proximal target cells with high efficiency. Indeed, exmiRNAs have been shown to be associated with pathological conditions such as cancer, proliferation, apoptosis, cellular development, cellular signaling, substance use disorder, and HIV pathogenesis [25,26,27,28]. However, the carriers of the pathological exmiRNAs remain unknown and some carriers of exmiRNA could masquerade as EVs when co-purified. 

In this study, we used the unparalleled nano-particle isolation tool—particle purification liquid chromatography (PPLC) [29] to separate EDTA blood plasma from SIV-infected RMs administered with cART, THC, or both, into paired EVs and ECs. We sequenced small RNAs of the paired EVs and ECs. This experimental design enabled a systematic investigation of the abundance and association of exmiRNAs with EVs and ECs and provided insight on how SIV infection or treatment with cART, THC, or both cART+THC regulate exmiRNA profiles. We showed that EV-associated miRNAs represent a minor portion of exmiRNAs. While a significant proportion (72.5%) of exmiRNAs were associated with ECs, cART, THC, or both did not change the abundance pattern of exmiRNAs within EVs and ECs. We showed that the administration of cART counteracted SIV-mediated reduction in EV-associated miR-206 and miR-378d counts, while administration of THC to SIV-infected RMs attenuated SIV-mediated downregulation of miR-99a-5p. Importantly, treatment of SIV-infected RMs with cART resulted in the longitudinal upregulation of 6 EV-associated miRNAs (miR-484, miR-107-3p, miR-206, miR-184, miR-1260b, miR-6132). Additionally, administration of both cART and THC to SIV-infected RMs longitudinally decreased 3 EV-associated miRNAs (miR-342-3p, miR-100-5p, miR181b-5p) and increased 3 EC-associated miRNAs (miR-676-3p, miR-574, miR-505-5p).

These data substantially improve our understanding of the effect of SIV infection or treatment with cART, THC, and cART+THC on the abundance and compartmentalization of exmiRNAs in EVs and ECs. 

## 2. Results

### 2.1. The Isolation Profiles of EDTA Blood Plasma exmiRNA Carriers (EVs and ECs) Are Different but Not Changed by SIV, THC, cART, or Both THC and cART Administered Together

**Figure 1:**
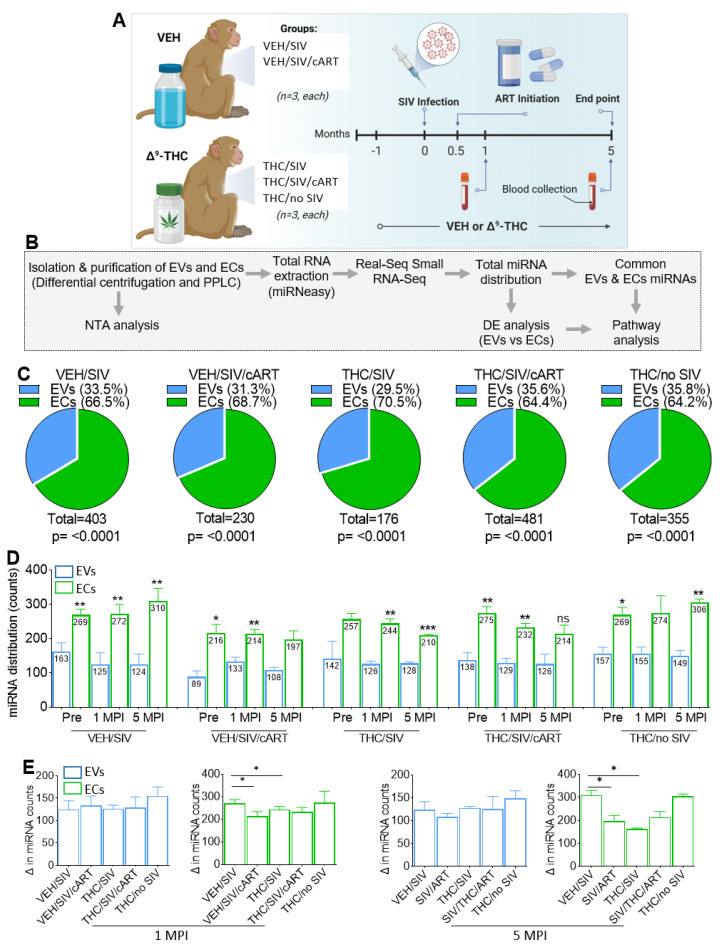
Study workflow for isolation and characterization of blood plasma derived EVs and ECs. (**A**) Description of experimental model. (**B**) Methodological workflow for isolation, and characterization of EVs and ECs. (**C**) Pie chart showing percent detectable miRNAs in the EVs and ECs for each treatment group. (**D**) The number of detectable miRNAs for the EVs and ECs at the Pre, 1 MPI and 5 MPI time points for VEH/SIV, VEH/SIV/ART, THC/SIV, THC/SIV/ART, and THC/no SIV treatment groups. (**E**) The number of detectable miRNAs for the EVs and ECs at 1 MPI (left) and 5 MPI (right) for VEH/SIV, VEH/SIV/ART, THC/SIV, THC/SIV/ART, and THC/no SIV treatment groups. To be included, miRNA count needed to be ≥1 for n = 3 RMs. Binary students’ t tests (Welch’s correction) were used to determine significant differences between EVs and ECs for each of the time points in each group. *** *p* < 0.005, ** *p* < 0.01, * *p* < 0.05, and ns = non-significant.

The study design stratified by treatment (SIV infection, THC, cART, and THC/cART treatments) is provided in Figure 1A and Table 1. In Table 2, we provided information on additional treatments that some of the RMs underwent. Note that the additional treatment (Table 2) did not affect the outcome of our study but was provided for full disclosure. The workflow for isolation of EVs and ECs as well as their characterization is shown in Figure 1B. Representative elution spectra for each treatment group with the locations of EVs and ECs are shown in Figure S1A. The spectral profiles, with the first peak (blue box) from fractions 66 to 80, denote regions enriched within EVs, and the last peak (green box) from fractions 233 to 264 denote regions enriched within ECs [29], with insets used to enlarge the distinct EV and EC profiles. The spectral profiles for EVs or ECs showed no significant variation among groups (Figure S1A). Nanoparticle tracking analysis (NTA) of EV size, concentration, and zeta-potential was conducted for VEH/SIV, VEH/SIV/cART, THC/SIV, THC/SIV/cART, and THC/no SIV treated RMs at 1 MPI and 5 MPI. On the average, EV size for VEH/SIV, VEH/SIV/cART, THC/SIV, THC/SIV/cART, and THC/no SIV at 1 MPI/5 MPI was 133.25/144.81 nm, 142.04/142.06 nm, 153.81/142.56 nm, 146.58/136.70 nm, and 138.05/134.21 nm, respectively (Figure S1B). The average EV concentration for VEH/SIV, VEH/SIV/cART, THC/SIV, THC/SIV/cART, and THC/no SIV at 1 MPI/5 MPI was 2.89e10/3.11e10 particles/mL, 2.00e10/2.40e10 particles/mL, 1.49e10/1.79e10 particles/mL, 1.72e10/2.50e10 particles/mL, 4.21e10/6.73e10 particles/mL, respectively (Figure S1C). On average, EV surface charge, measured as zeta-potential (ζ-potential) for VEH/SIV, VEH/SIV/cART, THC/SIV, THC/SIV/cART, and THC/no SIV at 1 MPI/5 MPI was −37.24/−35.76 mV, −36.63/−36.69mV, −43.79/−43.61 mV, −36.98/−32.24 mV, −37.31/−42.70 mV (Figure S1D). There was no significant effect of cART, THC, or THC/cART on EV size, concentration, and ζ-potential. It was noteworthy that EC size, concentration, and zeta-potential were not measured as the average EC particle size was lower than the 20 nm detection limit of the Zetaview PMX 110 with the standard laser configuration, therefore preventing the acquisition of accurate and reproducible data (https://www.excilone.com/client/document/particle-metrix--zetaview-brochure-0319_en_540.pdf, accessed on 20 November, 2022). However, analysis of protein concentrations of the EVs and ECs revealed differences in the concentrations of EVs and ECs within and across sample (Figure S1E).

### 2.2. Circulating miRNA Repertoire and Their Association with EVs and ECs following SIV Infection and Treatment 

In Manuscript 1 of this series, we showed that at 5 MPI, SIV infection altered the level of EV-associated miR-378d, miR-99a-5p, miR-206, miR-128a-3p, miR-128-b-3p and EC-associated miR-498, miR-671-5p, and miR-656-3p. To determine the effect of THC and cART on EV and EC miRNAome, blood plasma collected at 1 MPI and 5 MPI from RMs in Group 1 (VEH/SIV), Group 2 (THC/SIV), Group 3 (VEH/SIV/cART), Group 4 (THC /SIV/cART), and Group 5 (THC/no SIV) were used for the isolation of EVs and ECs via PPLC [29]. The paired EVs and ECs were subjected to sRNA-seq (Figure 1). 

Following RNA isolation, the yield and quality of RNA in each carrier were analyzed. Average EV and EC RNA yields (Figure S1E) and RNA quality (Figure S1F) at 1 MPI/5 MPI were variable but not significantly different for VEH/SIV, VEH/SIV/cART, THC/SIV, THC/SIV/cART, and THC/no SIV. Total RNA irrespective of time of infection ranged from 255 ng to 673 ng for EVs and 281 ng to 451 ng for ECs (Figure S1E). Likewise, RNA quality (A260/280 ratios) irrespective of time of infection ranged from 1.39 to 1.55 for EVs and 1.30 to 1.54 for ECs (Figure S1F). These data suggest that the total RNA content and quality were similar for EVs and ECs, although non-significant variabilities were observed at the individual animal level.

The miRNAome of the paired EVs and ECs were analyzed as we previously described [30]. Evaluation of cross-sectional changes in EVs and ECs miRNAome from all groups at 1 MPI and 5 MPI showed that the number of detectable miRNAs associated with EVs were significantly lower compared to those associated with ECs (Figure 1C). The differences in RNA content were irrespective of SIV infection and treatments (cART, THC, THC/cART). The percent of detectable miRNAs in the EV/EC fractions irrespective of length of infection (1 MPI, 5 MPI) were as follows: VEH/SIV: 33.5/66.5%; VEH/SIV/cART: 31.3/68.7%; THC/SIV: 29.5/70.5%; THC/SIV/cART: 35.6/64.4%; THC/no SIV: 35.8/64.2% (Figure 1C). At 1 MPI, the number of detectable miRNAs associated with EV/EC fractions across treatments were as follows: VEH/SIV: 125/272; VEH/SIV/cART 133/214, THC/SIV: 126/244 THC/SIV/cART 129/232, THC/no SIV: 155/274 (Figure 1D). Similarly, at 5 MPI, the number of detectable miRNAs associated with EV/EC fractions across treatments were as follows: VEH/SIV: 124/310; THC/SIV: 128/210, VEH/SIV/cART 108/197, SIV/cART/THC 126/214, THC/no SIV:149/306 (Figure 1D). In the VEH/SIV and THC/SIV groups, differences in detectable miRNAs in the EV and EC fractions were significant at both 1 MPI and 5 MPI. In the VEH/SIV/cART and THC/SIV/cART groups, differences in detectable miRNAs in the EV and EC fractions were significant at 1 MPI, while at 5 MPI detectable miRNAs were increased in the EC fraction but not the EV fraction, although the differences did not reach significance (Figure 1D). 

Further analysis suggested that miRNA counts did not change with increasing time of infection from 1 MPI to 5 MPI within each carrier (EVs and ECs). However, miRNA enrichment in EVs was stable compared to ECs (Figure 1E). While treatment had no effect on the number of detectable EV-associated miRNAs, THC and cART treatments, although not both (THC+cART) together, significantly decreased the number of EC-associated miRNAs at 1 MPI and 5 MPI (Figure 1E). 

### 2.3. Differences and Similarities in EV and EC Associated miRNA Repertoires following SIV Infection and Treatment with cART and THC

**Figure 2:**
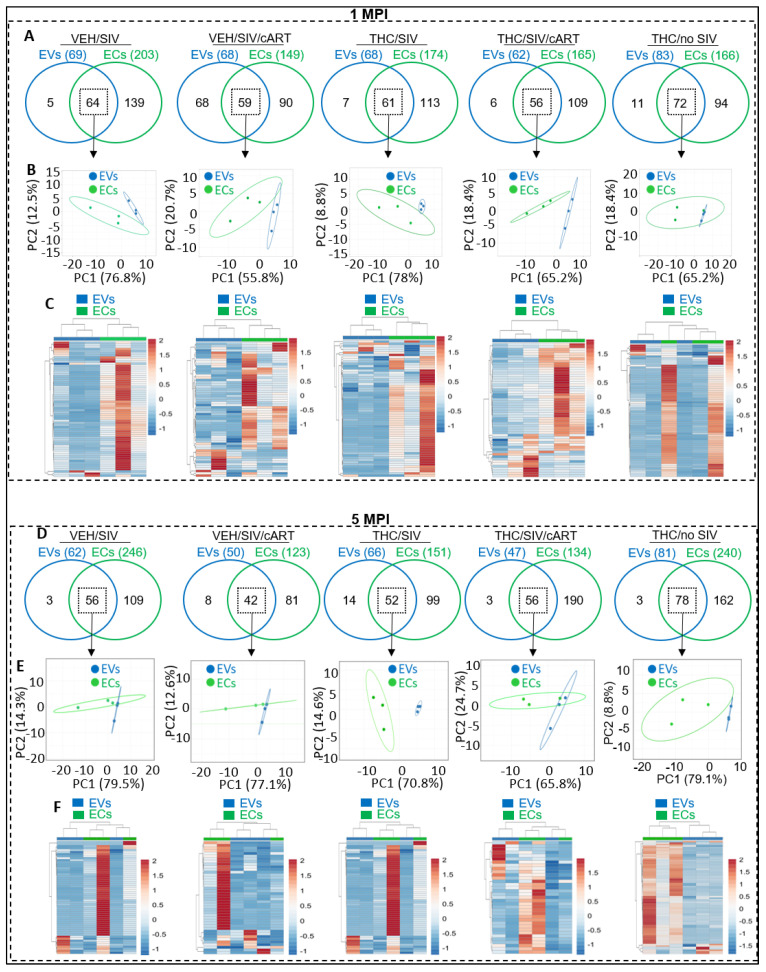
Repertoires of EV− and EC − associated miRNA following SIV infection and treatment with cART and THC. (**A**) Venn diagram comparing total detectable miRNAs for EVs and ECs at 1 MPI for VEH/SIV, VEH/SIV/ART, THC /SIV, THC/SIV /ART, and THC/no SIV treatment Groups. (**B**) PCA analysis of the common EVs and ECs miRNAs from the Venn diagrams (indicated by black square) at 1 MPI for the VEH/SIV, VEH/SIV/ART, THC/SIV, THC/SIV/ART, and THC/no SIV treatment groups. Unit variance scaling is applied to rows; SVD with imputation is used to calculate principal components. X and Y axis show principal component 1 and principal component 2. n = 3 RMs per treatment group. (**C**) Hierarchical clustering heatmap of the common EVs and ECs miRNAs from the Venn diagrams (indicated by black square) at 1 MPI for the VEH/SIV, VEH/SIV/ART, THC/SIV, THC/SIV/ART, and THC/no SIV treatment groups. Rows are centered; unit variance scaling is applied to rows. Both rows and columns are clustered using correlation distance and average linkage. N = 3 RMs per treatment group. (**D**) Venn diagram comparing total detectable miRNAs for EVs and ECs at 5 MPI for VEH/SIV, VEH/SIV/ART, THC/SIV, THC/SIV/ART, and THC/no SIV treatment groups. (**E**) PCA analysis of the common EV and associated miRNAs from the Venn diagrams (indicated by black square) at 5 MPI for the VEH/SIV, VEH/SIV/ART, THC/SIV, THC/SIV/ART, and THC/no SIV treatment groups. Unit variance scaling is applied to rows; SVD with imputation is used to calculate principal components. X and Y axis show principal component 1 and principal component 2. n = 3 RMs per treatment group. (**F**) Hierarchical clustering heatmap of the common EV and EC miRNAs from the Venn diagrams (indicated by black square) at 5 MPI for the VEH/SIV, VEH/SIV/ART, THC/SIV, THC/SIV/ART, and THC/no SIV treatment groups. Rows are centered; unit variance scaling is applied to rows. Both rows and columns are clustered using correlation distance and average linkage. n = 3 RMs per treatment group.

Here, we identified common miRNAs (defined as miRNAs associated with both EVs and ECs) and distinct miRNAs (defined as miRNAs associated with EVs and not ECs, and vice versa) to assess whether SIV, VEH/SIV/cART, THC/SIV, or THC/SIV/cART altered the profile of EV-associated or EC-associated miRNAs. As indicated by the two-way Venn diagrams, marked differences in the number of miRNAs associated with EVs versus ECs were detected at 1 MPI, with more miRNAs in ECs (Figure 2A). In the VEH/SIV group, the top 10 unique miRNAs by count in the EVs were miR-6132, miR-184, miR-542-3p, miR-875-5p, and miR-186-3p (Figure S2A, top), while miR- 223, miR-24-3p, miR-6529-5p, miR-382-5p, miR-154-5p, miR-484, miR-361-5p, miR-222-3p, miR-377-3p, and miR-18b were the top 10 miRNAs in ECs (Figure S2A, bottom). The SIV/cART group had 9 unique and 10 unique miRNAs, respectively, for EVs and ECs (Figure S2B), while the THC/SIV group had 7 unique and 10 unique miRNAs, respectively, for EVs and ECs (Figure S2C). Additionally, 6 unique and 10 unique miRNAs in EVs and ECs were identified in the THC/SIV/cART Group (Figure S2D), and the THC/no SIV group had 10 unique miRNAs in both EVs and ECs (Figure S2E). Despite the differences in the miRNA content of EVs and ECs, many miRNAs were associated with both EVs and ECs at 1 MPI, irrespective of treatment (Figure 2A, square with broken lines). PCA analysis of miRNAs shared between EVs and ECs at 1 MPI clearly showed that while common, the miRNA profiles differ between EVs and ECs across groups, with carrier-specific clustering observed (Figure 2B). Heatmap analysis further revealed that although the miRNAs were commonly detected in EVs and ECs, the intensities of their enrichment were different (Figure 2C). 

Similar to the observations made at 1 MPI, marked differences and similarities in miRNAs in each carrier—EVs versus ECs were detected at 5 MPI, with more miRNAs being associated with ECs across treatment groups (Figure 2D). While more miRNAs were associated with ECs, both EVs and ECs had many miRNAs in common, irrespective of treatment ( 2D–F). PCA plots of miRNAs shared between EVs and ECs at 5 MPI clearly showed that overall, the EV and EC miRNAs clustered separately across groups (Figure 2E). Additionally, heatmap analysis revealed the enrichment intensity of the common miRNAs between EVs and ECs (Figure 2F). In the VEH/SIV group, the top 10 unique miRNAs by count in the EVs were miR-185, miR-1260b, and miR-205 (Figure S2F, top), while miR-376b-3p, miR-376a-3p, miR-223, miR-146a-5p, miR-146b-4p, miR-6529-5p, miR-409-3p, miR-369-3p and miR-377-3p were the top 10 miRNAs in ECs (Figure S2F, bottom). The SIV/cART group had 8 unique and 10 unique miRNAs, respectively (Figure S2G), while the THC/SIV group had 10 unique miRNAs in both EVs and ECs (Figure S2H). Additionally, 10 unique miRNAs in EVs and ECs were significantly and differentially altered in the THC/SIV/cART group (Figure S2I), and the THC/no SIV group had 3 unique and 10 unique miRNAs in EVs and ECs, respectively (Figure S2J). Together, these data are consistent with reports showing that exRNAs are associated with various circulating carriers, including lipoproteins [31,32], EVs [29,33], and membraneless condensates (MCs) [29], also known as extracellular condensates (ECs). 

Three miRNAs with the highest counts in each carriers are listed in Table 3. Large differences were observed between EVs and ECs at different times post infection and across the treatment groups. The vertebrate genomes contain about 12 let-7 family members (let-7a-1, -2, -3; let-7b; let-7c; let-7d; let-7e; let-7f-1, -2; let-7g; let-7i; miR-98) with identical seed sequence. Of these, let-7a-5p and let-7c-5p were amongst the EV-associated miRNAs with the highest counts, along with miR-26a-5p. These 3 miRNAs were present in EVs across all treatments and at different time points, albeit at different levels (Table 3). In contrast to EVs, miR-27a-3p is the one miRNA that is present in ECs at all time points and across treatments, except at 5 MPI of VEH/SIV/cART group and Pre of THC/SIV/cART group (Table 3). These data suggest possible preferential enrichment of specific miRNA with different carriers, as was also suggested by Arroyo et al., who showed that Let-7a is exclusively associated with EVs [34].

### 2.4. Varying miRNA Abundance in EVs and ECs in Response to SIV Infection and Viral Suppression by ART

In Manuscript 1 of this series, we showed that at 5 MPI, SIV infection altered the level of EV-associated miR-378d, miR-99a-5p, miR-206, miR-128a-3p, miR-128-b-3p and EC-associated miR-498, miR-671-5p, and miR-656-3p. Here, we used samples from Group 1 and Group 3 (Table 1) to assess the effects of cART on miRNAome of EVs and ECs. 

**Figure 3:**
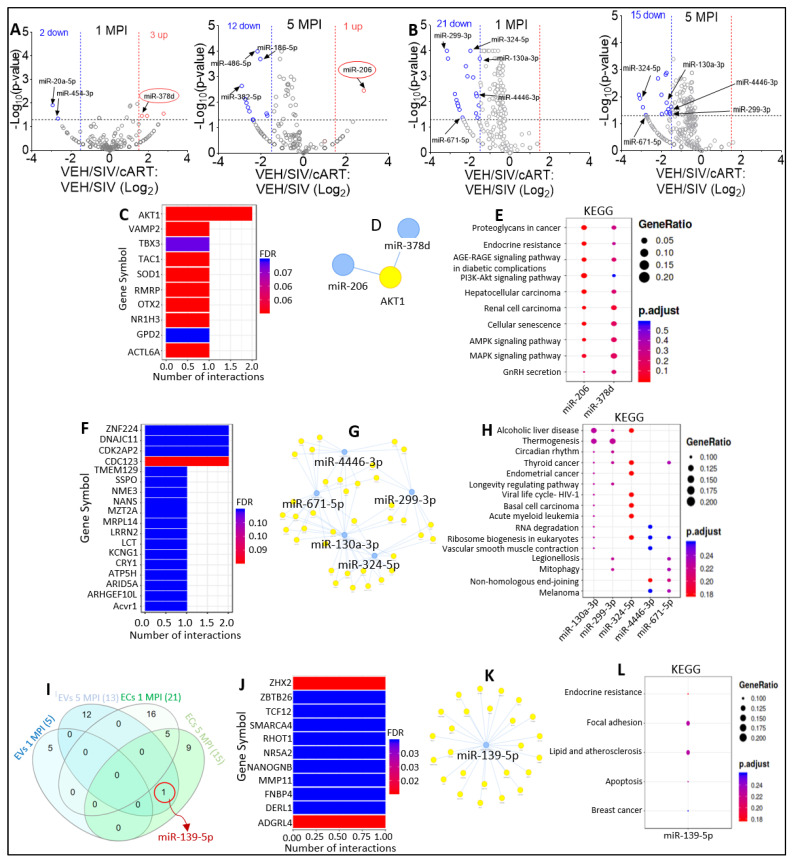
Varying miRNA abundance in EVs and ECs in response to SIV infection and cART. (**A**) Volcano plot showing down-(blue) regulated and up-(red) regulated miRNAs in VEH/SIV/ART EVs relative to VEH/SIV EVs at 1 MPI (left) and 5 MPI (right) as shown by black arrows. Red circle indicates miRNAs (miR-378d, miR-206) suppressed by SIV infection in Figs. 5C, 5E of Manuscript 1. (**B**) Volcano plot showing down-(blue) regulated miRNAs in SIV/ART ECs relative to SIV ECs at 1 MPI (left) and 5 MPI (right). Black arrows indicate miRNAs shown to be significantly downregulated at 1 MPI and 5 MPI. (**C**) miRNA-target enrichment analysis showing top target genes by # of interactions for miR-206 and miR-378d. (**D**) Visualization of miRNA-target interaction network for miR-206 and miR-378d. The blue circles represent the miRNAs, while the target genes are represented by the yellow circles. Pathways are represented by blue lines. (**E**) Dot plot of functional enrichment analysis for target genes of miR-206 and miR-378d. Color of dots represent adjusted p-values (FDR) and size of dots represents gene ratio (number of miRNA targets found enriched in each category/number of total genes associated to that category. (**F**) miRNA-target enrichment analysis showing top target genes by number of interactions for the 5 EC-associated miRNAs significantly downregulated at 1 MPI and 5 MPI; miR-299-3p, miR-324-5p, miR-4446-3p, miR-299-3p, and miR-671-5p. (**G**) Visualization of miRNA-target interaction network for miR-299-3p, miR-324-5p, miR-4446-3p, miR-299-3p, and miR-671-5p. The blue circles represent the miRNAs, while the target genes are represented by the yellow circles. Pathways are represented by blue lines. (**H**) Dot plot of functional enrichment analysis for target genes of miR-299-3p, miR-324-5p, miR-4446-3p, miR-299-3p, and miR-671-5p. Color of dots represent adjusted p-values (FDR) and size of dots represents gene ratio (number of miRNA targets found enriched in each category/number of total genes associated with that category). (**I**) Venn diagram comparing significantly modulated miRNAs in EVs and ECs at 1 MPI and 5 MPI. Red circle indicates miRNA (miR-139-5p) that was found to be significantly downregulated at 5 MPI in both EVs and ECs. (**J**) miRNA-target enrichment analysis showing top target genes by number of interactions for miR-139-5p. (**K**) Visualization of miRNA-target interaction network for miR-139-5p. The blue circles represent the miRNAs, while the target genes are represented by the yellow circles. Pathways are represented by blue lines. (**L**) Dot plot of functional enrichment analysis for miR-139-5p. Color of dots represent adjusted p-values (FDR) and size of dots represent gene ratio (number of miRNA targets found enriched in each category/number of total genes associated to that category).

Comparison of EV miRNAome for VEH/SIV versus VEH/SIV/cART at 1 MPI showed that in the presence of cART, 2 miRNAs (miR-454-3p and miR-20a-5p) were significantly downregulated, while 3 miRNAs (miR-378d, miR-1260b, miR-143-3p) were significantly upregulated (Figure 3A, left). At 5 MPI, cART treatment of SIV-infected RMs resulted in significant downregulation of 12 miRNAs (miR-382-5p, miR-187-3p, miR-652, miR-409-3p, miR-139-5p, miR-222-3p, miR-423-3p, miR-486-3p, miR-486-5p, miR-186-5p, miR-28-3p, and miR-10b-5p), while the muscle-specific miR-206 that was significantly decreased by SIV was significantly increased by cART (Figure 3A, right). Interestingly, Simon et al. 2017 showed that SIV decreased miR-206 expression in myoblasts, which may contribute to decreased myogenic differentiation potential in chronic binge alcohol administered SIV-infected RMs [35]. Based on these data, it is plausible that treatment with cART may counteract SIV-mediated reduction in miR-206 and miR-378d counts. With respect to EC miRNAome, volcano plot analysis of SIV versus SIV/cART miRNAome show that treatment with cART resulted in significant downregulation of 21 miRNAs at 1 MPI (Figure 3B, left) and 15 miRNAs at 5 MPI (Figure 3B, right). Interestingly, cART treatment decreased EC-associated miR-299-3p, miR-324-5p, miR-4446-3p, miR- 299-3p, and miR-671-5p both at 1 MPI and 5 MPI (Figure 3B black arrows). 

Insight into the potential functions of EV-associated SIV-downregulated miR-206 and miR-378d that were upregulated by cART treatment was provided by functional-enrichment analysis using MIENTURNET miRTarbase. The top 10 most significant target genes of miR-206 and miR-378d include AKT1, VAMP2, TBX3, TAC1, SOD1, RMRP, OTX2, NR1H3, GPD2, ACTL6A (Figure 3C), and miRNA-target interaction networks further identified AKT1 as being targeted by both miRNAs (Figure 3D). Interestingly, AKT signaling plays an important role in HIV pathogenesis by hyperactivating T cells [36], and HIV protease inhibitors blocked both AKT activation and HIV reactivation in myeloid cells [37]. KEGG pathway analysis showed that miR-206 and miR-378d and their target genes were associated with proteoglycans in cancer, endocrine resistance, AGE-RAGE signaling pathway in diabetic complications, PI3K-Akt signaling pathway, hepatocellular carcinoma, renal cell carcinoma, cellular senescence, AMPK and MAPK signaling pathways, and GnRH secretion (Figure 3E). 

Similarly, the potential functions of EC-associated miR-299-3p, miR-324-5p, miR-4446-3p, miR-299-3p, and miR-671-5p were assessed. The top 10 most significant target genes of EC-associated SIV-upregulated miR-299-3p, miR-324-5p, miR-4446-3p, miR- 299-3p, and miR-671-5p that were downregulated by cART treatment included: ZNF224, DNAJC11, CDK2AP2, CDC123, TMEM129, SSPO, NME3, NANS, MZT2A, and MRPL14 (Figure 3F). MiRNA-target interaction networks depicted complex connections between the miRNAs and their predicted genes (Figure 3G). KEGG pathway predicted that the target genes of miR-299-3p, miR-324-5p, miR-4446-3p, miR-299-3p, and miR-671-5p were associated with: alcoholic liver disease, thermogenesis, circadian rhythm, thyroid cancer, endometrial cancer, longevity regulating pathway, viral life cycle—HIV-1, basal cell carcinoma, acute myeloid leukemia, and RNA degradation (Figure 3H).

Furthermore, InteractiVenn analysis identified miR-139-5p as commonly downmodulated by cART in EVs and ECs at 5 MPI (Figure 3I). MIENTURNET miRTarbase analysis identified ZHX2, ZBTB26, TCF12, SMARCA4, RHOT1, NRFA2, NANOGNB, MMP11, FNBP4, and DERL1 as the top 10 most significant target genes of miR-139-5p (Figure 3J) with predicted gene networks shown in Figure 3K. KEGG pathway analysis suggested that the target genes of miR-139-5p are associated with endocrine resistance, focal adhesion, lipid and atherosclerosis, apoptosis, and breast cancer (Figure 3L).

### 2.5. Effect of THC on miRNA Profiles following SIV Infection in the Absence of Viral Suppression by cART

Here, we sought to determine the effects of THC on EVs and ECs miRNAome from viremic animals. For this purpose, paired EVs and ECs isolated from blood plasma collected at 1 MPI and 5 MPI from RMs in Group 1 (VEH/SIV) and Group 2 (THC/SIV) were analyzed (Figure 1). 

**Figure 4:**
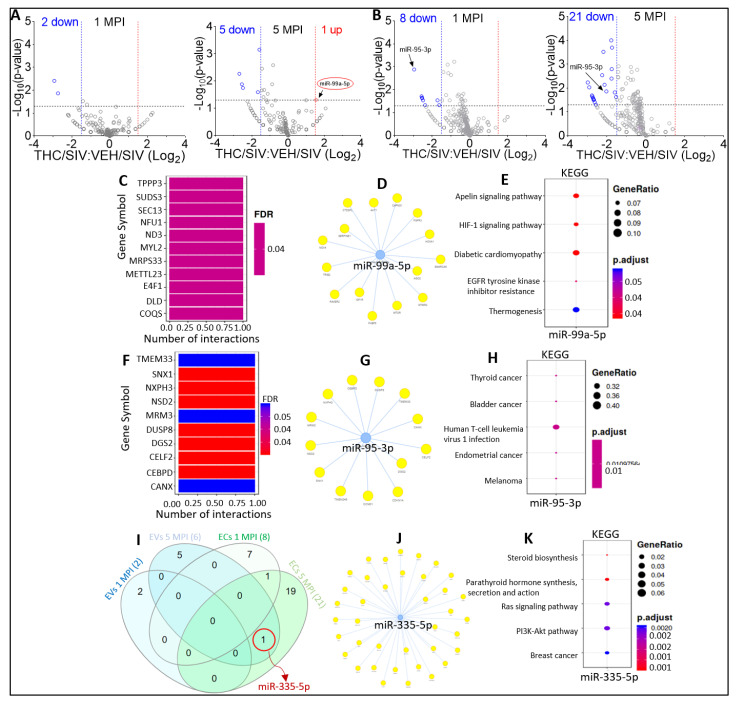
Alterations in miRNA profile of THC-treated SIV-infected cART-naive RMs. (**A**) Volcano plot showing down-(blue) regulated and up-(red) regulated miRNAs in THC/SV EVs relative to VEH/SIV EVs at 1 MPI (left) and 5 MPI (right). Red circle indicates miRNAs suppressed by SIV infection miR-99a-5p. (**B**) Volcano plot showing down-(blue) regulated and miRNAs in THC/SIV ECs relative to SIV ECs at 1 MPI left) and 5 MPI (right). Black arrows indicate miRNAs shown to be significantly downregulated at both MPI and 5 MPI. (**C**) miRNA-target enrichment analysis showing top target genes by number of interactions for miR-99a-5p. (**D**) Visualization of miRNA-target interaction network for miR-9a-5p. The blue circles represent the miRNAs, while the target genes are represented by the yellow circles. Pathways are represented by blue lines. (**E**) Dot plot of functional enrichment analysis for target genes of miR-99a-5p. Color of dots represent adjusted p-values (FDR) and size of dots represents gene ratio (number of miRNA targets found enriched in each category/number of total genes associated to that category. (**F**) miRNAtarget enrichment analysis showing top target genes by number of interactions for the EC-associated miRNA (miR-5-3p) significantly downregulated at 1 MPI and 5 MPI. (**G**) Visualization of miRNA-target interaction network for miR-95-3p. The blue circles represent the miRNAs, while the target genes are represented by the yellow circles. Pathways are represented by blue lines. (**H**) Dot plot of functional enrichment analysis for target genes of miR-95-3p. Color of dots represent adjusted p-values (FDR) and size of dots represents gene ratio (number of miRNA targets found enriched in each category number of total genes associated with that category. (**I**) Venn diagram comparing significantly modulated miRNAs in the EVs and ECs at MPI and 5 MPI. Red circle indicates miRNA (miR-335-5p) that was found to be significantly downregulated at 5 MPI in both EVs and ECs. (**J**) Visualization of miRNAtarget interaction network for miR-335-5p. (**K**) Dot plot of functional enrichment analysis for miR-335-5p Color of dots represent adjusted p-values (FDR) and size of dots represents gene ratio (number of miRNA targets found enriched in each category number of total genes associated to that category.

**Figure 5:**
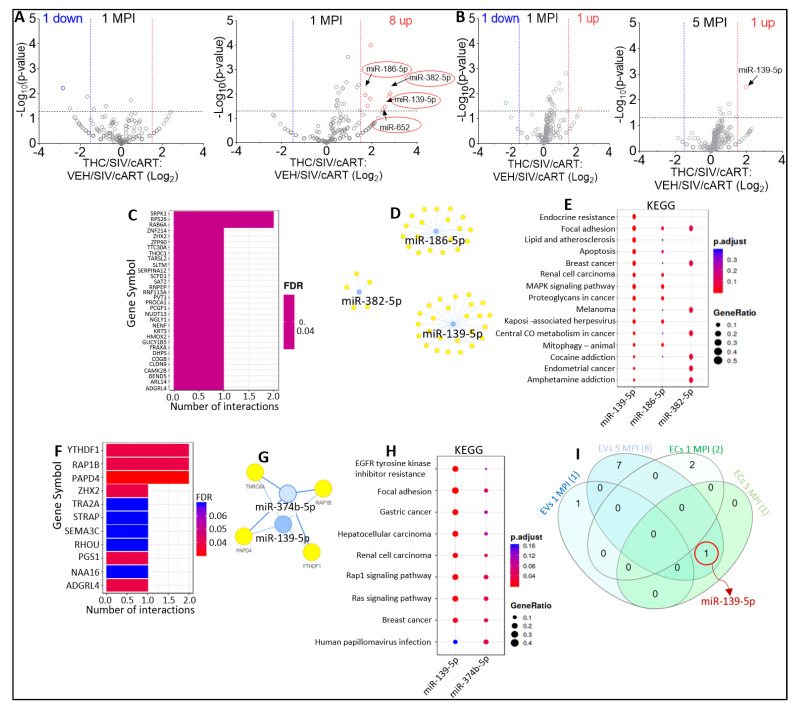
Alterations in miRNA profile of THC-treated SIV-infected cART-treated RMs. (**A**) Volcano plot showing down-(blue) regulated and up-(red) regulated miRNAs in THC/SIV/ART EVs relative to VEH/SIV/ART EVs at 1 MPI (left) and 5 MPI (right). Red circle indicates miRNAs (miR-186-5p, miR-382-5p, miR-139-5p, and miR-652) suppressed by ART treatment of SIV-infected macaques. (**B**) Volcano plot showing down-(blue) regulated and miRNAs in THC/SIV/ART ECs relative to VEH/SIV/ART ECs at 1 MPI (left) and 5 MPI (right). (**C**) miRNA-target enrichment analysis showing top target genes by number of interactions for miR-186-5p, miR-382-5p, miR-139-5p. (**D**) Visualization of miRNA-target interaction network for miR-574, miR-374b-5p and miRA-139-5p. The blue circles represent the miRNAs, while the target genes are represented by the yellow circles. Pathways are represented by blue lines. (**E**) Dot plot of functional enrichment analysis for target genes of miR-186-5p, miR-382-5p, miR-139-5p. Color of dots represent adjusted p-values (FDR) and size of dots represents gene ratio (number of miRNA targets found enriched in each category/number of total genes associated to that category. (**F**) miRNA-target enrichment analysis showing top target genes by number of interactions for miR-139-5p and miR-374b-5p. (**G**) Visualization of miRNA-target interaction network for miR-139-5p and miR-374b-5p. The blue circles represent the miRNAs, while the target genes are represented by the yellow circles. Pathways are represented by blue lines. (**H**) Dot plot of functional enrichment analysis for target genes of miR-139-5p and miR-374b-5p. Color of dots represent adjusted p-values (FDR) and size of dots represents gene ratio (number of miRNA targets found enriched in each category/number of total genes associated to that category. (**I**) Venn diagram comparing significantly modulated miRNAs in EVs and ECs at 1 MPI and 5 MPI. Red circle indicates miR-139-5p that was significantly downregulated at 5 MPI in both EVs and ECs.

Volcano plot analysis of EV miRNAome for VEH/SIV versus THC/SIV showed that in the presence of THC at 1 MPI, 2 miRNAs (miR-425, miR-20a-5p) were significantly downregulated (Figure 4A, left). At 5 MPI, THC treatment resulted in the significant downregulation of 5 EV-associated miRNAs (miR-106b-5p, miR-652, miR-139-5p, miR-98, miR-335-5p) and a significant increase in the counts of miR-99a-5p (Figure 4A, right, red oval). Interestingly, miR-99a-5p was amongst the miRNA significantly decreased in EVs of SIV-infected RMs at 5 MPI, as demonstrated in Figure 5E of Manuscript 1 of this series. THC treatment had no significant effect on the other SIV-downregulated miRNAs at 1 MPI and 5 MPI. These data suggest that THC may attenuate SIV-mediated downregulation of miR-99a-5p. With respect to EC miRNAome, volcano plot analysis of VEH/SIV versus THC/SIV miRNAome showed that treatment with THC resulted in significant downmodulation of 8 (miR-656-3p, miR-95-3p, miR-32-5p, miR-148b-3p, miR-214-3p, miR-188-5p, miR-505-3p, miR-215-5p) at 1 MPI (Figure 4B, left) and 21 (miR-574, miR-7195-3p, miR-671-5p, miR-330-3p, miR-496, miR-382-3p, miR-199a-5p, miR-199a, let-7e-5p, miR-494-3p, miR-193a-5p, miR-19a-3p, miR-378a, miR-21-3p, miR-95-3p, miR-335-5p, miR-150-5p, miR-7, miR-19b, miR-4446-3p, miR-539) miRNAs at 5 MPI (Figure 4B, right). Interestingly, THC treatment downmodulated EC-associated miR-95-3p both at 1 MPI and 5 MPI (Figure 4B, black arrows).

Functional-enrichment analysis identified TPPP3, SUDS3, SEC13, NFU1, ND3, MYL2, MRPS33, METTL23, E4F1, and DLD as the top 10 most significant target genes of miR-99a-5p (Figure 4C), with the miRNA-target interaction networks (Figure 4D) showing the connections between the miRNAs and their predicted genes. KEGG pathway analysis showed that the target genes of miR-99a-5p were associated with: apelin signaling pathway, HIF-1 signaling pathway, diabetic cardiomyopathy, EGFR tyrosine kinase inhibitor resistance, and thermogenesis (Figure 4E).

In ECs, TMEM33, SNX1, NXPH3, NSD2, MRM3, DUSP8, DGS2, CELF2, CEBPD, and CANX were identified as the top 10 most significant target genes of miR-95-3p (Figure 4F), with the miRNA-target interaction network (Figure 4G) showing the connections between miR-95-3p and predicted target genes. The target genes of miR-95-3p were predicted to be associated with: thyroid cancer, bladder cancer, human T-cell leukemia virus 1 infection, endometrial cancer, and melanoma (Figure 4H).

To identify THC-altered miRNAs shared between EVs and ECs, we fed InteractiVenn with the miRNAs that were differentially associated with EVs and ECs. We identified miR-335-5p as the only miRNA significantly downregulated by THC treatment in both EVs and ECs at 5 MPI (Figure 4I). Target interaction network analysis of miR-335-5p depicts the connections between miR-335-5p and its predicted gene networks (Figure 4J). KEGG pathway analyses showed that miR-335-5p target genes are predicted to associate with: Steroid biosynthesis, Parathyroid hormone synthesis, secretion and action, Ras signaling pathway, PI3K-Akt signaling pathway, and breast cancer (Figure 4K).

### 2.6. Effect of THC on miRNA Profile of SIV-infected Rhesus Macaques under Suppressive cART 

To determine the specific effect of THC on EVs and ECs miRNAome of virally suppressed SIV-infected RMs, we used EVs and ECs isolated from the blood plasma collected at 1 MPI and 5 MPI from RMs in Group 3 (SIV/ART) and Group 4 (THC /SIV/ART) as described in Table 1. 

EV miRNAome of virally suppressed animals without THC (SIV/cART) or treated with THC (THC/SIV/cART) showed that in the presence of THC at 1 MPI, one EV-associated miRNA (miR-6529-5p) was significantly downregulated (Figure 5A, left). At 5 MPI, THC treatment resulted in the significant upregulation of eight EV-associated (miR-10a-5p, miR-657, miR-29c-3p, miR-140-5p, miR-186-5p, miR-382-5p, miR-139-5p, and miR-652) miRNAs (Figure 5A, right). These data indicate that at 5 MPI THC treatment may attenuate cART induced downregulation of miR-186-5p, miR-382-5p, miR-139-5p, and miR-652. With respect to EC miRNAome, volcano plot analysis of VEH/SIV/cART versus THC/SIV/cART showed that in the presence of THC at 1 MPI, one miRNA (miR-574) was significantly downregulated, while one miRNA (miR-374b-5p) was significantly upregulated (Figure 5B, left). At 5 MPI, THC treatment resulted in significant increase in the count of EC-associated miR-139-5p (Figure 5B, right). 

Functional-enrichment analysis identified SRPK1, RPS26, RAB6A, ZNF214, ZHX2, ZFP90, TTC30A, THOC1, TARSL2, SLTM as the top 10 most significant target genes of miR-186-5p, miR-382-5p, miR-139-5p (Figure 5C). For this analysis, miR-652 was excluded because it was not found in the miRTarbase database, used by MIENTURNET and DIANA. Visualization of the EVs miRNA-target interaction networks depicted the connections between the miRNAs and the predicted target genes (Figure 5D). According to KEGG pathway analysis, the target genes of miR-186-5p, miR-382-5p, and miR-139-5p were predicted to be associated with endocrine resistance, focal adhesion, lipid and atherosclerosis, apoptosis, breast cancer, renal cell carcinoma, MAPK signaling pathway, proteoglycans in cancer, melanoma, Kaposi sarcoma-associated herpesvirus, as well as cocaine and amphetamine addiction (Figure 5E).

For additional insight into the functions of miR-139-5p and miR-374b-5p, both of which were significantly upregulated in ECs following THC treatment, we performed functional-enrichment analysis. The top 10 most significant target genes of miR-139-5p and miR-374b-5p include: YTHDF1, RAP1B, PAPD4, ZHX2, TRA2A, STRAP, SEMA3C, RHOU, PGS1, NAA16, and ADGRL4 (Figure 5F). Visualization of the miRNA-target interaction networks showed complex connections between the miRNAs and their predicted genes (Figure 5G). KEGG pathway analysis suggested that the target genes of miR-139-5p and miR-374b-5p were associated with: EGFR tyrosine kinase inhibitor resistance, Focal adhesion, Gastric cancer, Hepatocellular carcinoma, Renal cell carcinoma, Rap1 signaling pathway, Ras signaling pathway, Breast cancer, and Human papillomavirus infection. and Human papillomavirus infection (Figure 5H). Interestingly, similar to the observation in Figure 3I, miR-139-5p was present in both EVs and ECs at 5 MPI (Figure 5I).

### 2.7. Effect of cART on miRNA Profile of SIV-infected Rhesus Macaque in the Presence of Anti-Inflammatory THC 

To assess the effects of cART in EV and EC miRNAome in the presence of THC, we used EVs and ECs isolated from the blood plasma collected at 1 MPI and 5 MPI from RMs in Group 2 (THC/SIV) and Group 4 (THC /SIV/cART) (Table 1). 

**Figure 6:**
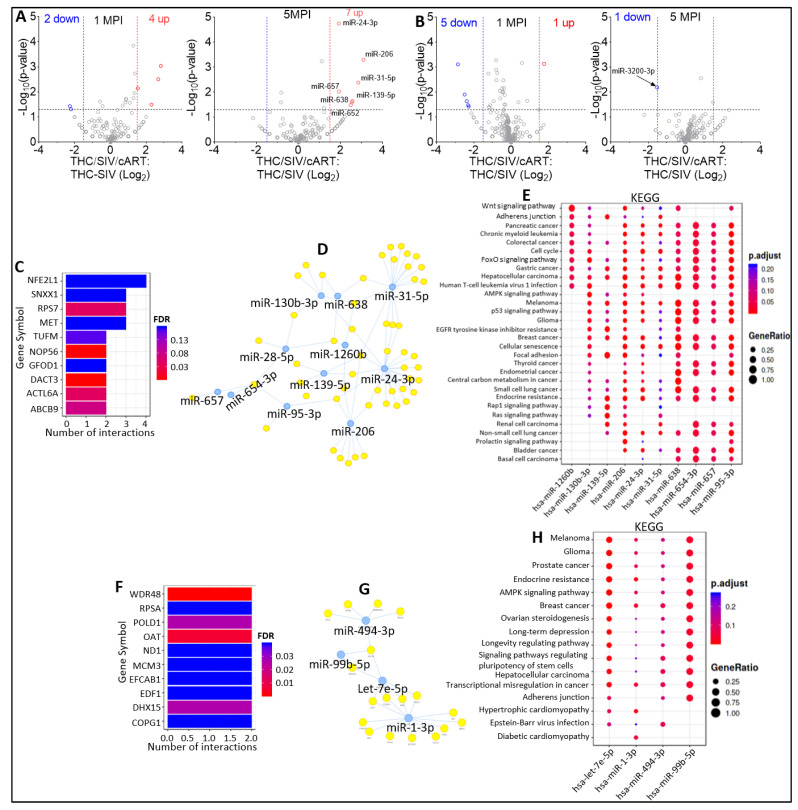
Alterations in miRNA profile of THC-treated, SIV-infected, cART-treated RMs. (**A**) Volcano plot showing down-(blue) regulated and up-(red) regulated miRNAs in THC/SIV/ART EVs relative to THC/SIV EVs at 1 MPI (left) and 5 MPI (right). (**B**) Volcano plot showing down-(blue) regulated and miRNAs in THC/SIV/ART ECs relative to THC/SIV ECs at 1 MPI (left) and 5 MPI (right). (**C**) miRNA-target enrichment analysis showing top target genes by number of interactions for the 13 significantly modulated EV-associated miRNAs (miR-130b-3p, miR-654-3p, miR-28-5p, miR-95-3p, miR-144, miR-1260b, miR-24-3p, miR-206, miR-31-5p, miR-657, miR-139-5p, miR-638, miR-652) at 1 MPI and 5 MPI. (**D**) Visualization of miRNA-target interaction network for the 13 significantly modulated EV-associated miRNAs. The blue circles represent the miRNAs, while the target genes are represented by the yellow circles. Pathways are represented by blue lines. (**E**) Dot plot of functional enrichment analysis for the 13 significantly modulated EV miRNAs. Color of dots represent adjusted p-values (FDR) and size of dots represents gene ratio (number of miRNA targets found enriched in each category/number of total genes associated to that category. (**F**) miRNA-target enrichment analysis showing top target genes by number of interactions for the 7 EC-associated miRNA significantly modulated at 1 MPI and 5 MPI. (**G**) Visualization of miRNA-target interaction network for the 7 EC-associated miRNA significantly modulated at 1 MPI and 5 MPI. The blue circles represent the miRNAs, while the target genes are represented by the yellow circles. Pathways are represented by blue lines. (**H**) Dot plot of functional enrichment analysis for target genes of the 7 EC-associated miRNA significantly modulated at 1 MPI and 5 MPI. Color of dots represent adjusted p-values (FDR) and size of dots represents gene ratio (number of miRNA targets found enriched in each category/number of total genes associated to that category.

Volcano plot analysis of THC/SIV/cART versus THC/SIV EV miRNAome showed that in the presence of cART at 1 MPI, 2 miRNAs were significantly downregulated, and 4 miRNAs (miR-28-5p, miR-95-3p, miR-144, miR-1260b) were significantly upregulated (Figure 6A, left). At 5 MPI, cART treatment resulted in the significant downregulation of 7 EV-associated (miR-24-3p, miR-206, miR-31-5p, miR-657, miR-139-5p, miR-638, miR-652) miRNAs (Figure 6A, right). Interestingly, treatment with THC also resulted in significant decrease in miR-139-5p and miR-652 in viremic RMs (Figure 4A). These data indicate that cART treatment may attenuate THC induced downregulation of miR-139-5p and miR-652. cART treatment had no significant effect on the other 4 miRNAs (miR-106b-5p, miR-98, miR-335-5p, miR-99a-5p) significantly downmodulated by THC. With respect to EC miRNAome, volcano plot analysis of THC/SIV/cART versus THC/SIV showed that in the presence of cART at 1 MPI, 5 miRNAs (miR-99b-5p, let-7e-5p, miR-494-3p, miR-628-3p, miR-605) were significantly downregulated, and 1 miRNA (miR-1-3p) was significantly upregulated (Figure 6B, left). At 5 MPI, cART treatment resulted in significant decrease in the counts of EC- associated miR-3200-3p (Figure 6B, right). 

Further analysis of the 13 EV-associated miRNAs significantly modulated by cART identified NFE2L1, SNX1, RPS7, MET, TUFM, NOP56, GFOD1, DACT3, ACTL6A, and ABCB9 as the top 10 most significant target genes (Figure 6C). Visualization of the miRNA-target interaction networks showed complex connections between the miRNAs and their predicted genes (Figure 6D). KEGG pathway analysis showed that the target genes of the 13 EV-associated miRNAs significantly modulated by cART were associated with: Wnt signaling pathway, adherens junction, pancreatic cancer, chronic myeloid leukemia, colorectal cancer, cell cycle, FoxO signaling pathway, gastric cancer, hepatocellular carcinoma, and human T-cell leukemia virus 1 infection (Figure 6E). 

For potential insight into the 7 EC-associated miRNAs significantly modulated by cART, we performed functional-enrichment analysis and identified WDR48, RPSA, POLD1, OAT, ND1, MCM3, EFCAB1, EDF1, DHX15, and COPG1 as the top 10 most significant target genes (Figure 6F). Visualization of the EC-associated miRNA-target interaction networks depicted complex connections between the miRNAs and their predicted genes (Figure 6G). KEGG pathway analysis suggested that the target genes of the 7 EC-associated miRNAs significantly modulated by cART are associated with: melanoma, glioma, prostate cancer, endocrine resistance, AMPK signaling pathway, breast cancer, ovarian steroidogenesis, long-term depression, longevity regulating pathway, and signaling pathways regulating pluripotency of stem cells (Figure 6H).

### 2.8. Defining Clinically Relevant miRNAs through Bioinformatics Integrative Analysis of Longitudinal Changes in the miRNAome of RM EVs and ECs Induced by SIV, cART and/or THC 

**Figure 7:**
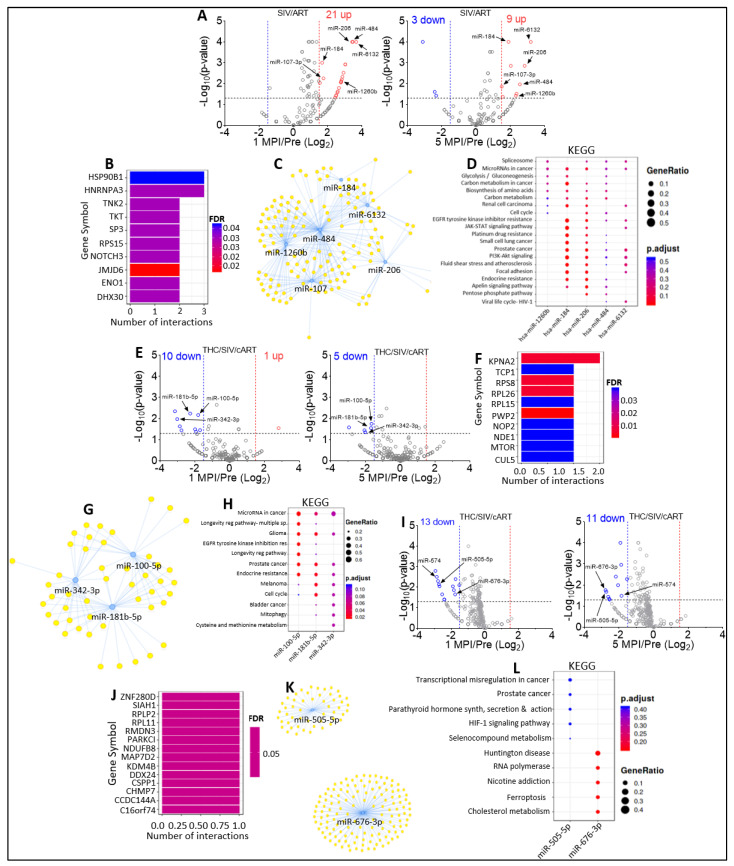
Identification of differentially and longitudinally altered EV − and EC − associated miRNAs of SIV-infected cART and/or THC treated RMs: (**A**) Volcano plot showing down-(blue) regulated and up-(red) regulated miRNAs in SIV/ART EVs at 1 MPI (left) and at 5 MPI (right). Black arrows indicate longitudinally upregulated miRNAs. (**B**) miRNA-target enrichment analysis showing top target genes by number of interactions for the longitudinally upregulated EV-associated miRNAs in panel A. (**C**) Visualization of miRNA-target interaction network for the longitudinally upregulated EV-associated miRNAs in panel A. The blue circles represent the miRNAs, while the target genes are represented by the yellow circles. Pathways are represented by blue lines. (**D**) Dot plot of functional enrichment analysis for the longitudinally upregulated EV-associated miRNAs in panel A. Color of dots represent adjusted p-values (FDR) and size of dots represents gene ratio (number of miRNA targets found enriched in each category/number of total genes associated to that category). (**E**) Volcano plot showing down-(blue) regulated and up-(red) regulated miRNAs in THC/SIV/ART EVs at 1 MPI (left) and at 5 MPI (right). Black arrows indicate longitudinally THC/ART-downregulated miRNAs. (**F**) miRNA-target enrichment analysis showing top target genes by number of interactions for the longitudinally downregulated EV-associated miRNAs in panel E. (**G**) Visualization of miRNA-target interaction network for the longitudinally downregulated EV-associated miRNAs in panel E. The blue circles represent the miRNAs, while the target genes are represented by the yellow circles. Pathways are represented by blue lines. (**H**) Dot plot of functional enrichment analysis for the longitudinally downregulated EV-associated miRNAs in panel E. Color of dots represent adjusted p-values (FDR) and size of dots represents gene ratio (number of miRNA targets found enriched in each category/number of total genes associated to that category. (**I**) Volcano plot showing down-(blue) regulated and up-(red) regulated miRNAs in THC/SIV/ART ECs at 1 MPI (left) and at 5 MPI (right). Black arrows indicate longitudinally THC/ART-downregulated miRNAs. (**J**) miRNA-target enrichment analysis showing top target genes by number of interactions for the longitudinally downregulated EC-associated miRNAs in panel I. (**K**) Visualization of miRNA-target interaction network for the longitudinally downregulated EC-associated miRNAs in panel I. The blue circles represent the miRNAs, while the target genes are represented by the yellow circles. Pathways are represented by blue lines. (**L**) Dot plot of functional enrichment analysis for the longitudinally downregulated EC-associated miRNAs in panel I. Color of dots represent adjusted p-values (FDR) and size of dots represents gene ratio (number of miRNA targets found enriched in each category/number of total genes associated to that category.

As well as the cross-sectional analysis of the miRNA changes in EVs and ECs across all study groups, our study design also allowed the analysis of longitudinal changes in miRNAome from 1 MPI to 5 MPI. Volcano plot analysis of EVs isolated from SIV-infected RMs treated with cART (VEH/SIV/cART) showed a significant upregulation of 21 miRNAs (miR-484, miR-7, miR-143-3p, miR-222-3p, miR-99a-5p, miR-486-5p, miR-140-5p, miR-125b-5p, miR-107-3p, miR-122a-5p, miR-378d, miR-206, miR-184, miR-30d-5p, miR-769-5p, miR-93-5p, miR-1260b, miR-138-5p, miR-205, miR-29a-3p, miR-6132) at 1 MPI (Figure 7A, left). At 5 MPI, cART treatment resulted in the significant downregulation of 3 miRNAs (miR-498, miR-144, miR-1249) and the significant upregulation of 9 (miR-1-3p, miR-484, miR-429-3p, miR-107, miR-206, miR-184, miR-1260b, miR-361-5p, miR-6132) miRNAs (Figure 7A, right). Interestingly, 6 miRNAs, including miR-484, miR-107, miR-206, miR-184, miR-1260b, and miR-6132 were longitudinally and significantly upregulated in EVs by cART (Figure 7A, black arrows). The magnitude of longitudinal changes in the different EV-associated miRNAs is shown in Table 4. 

MIENTURNET miRTarbase predicted HSP90B1, HNRNPA3, TNK2, TKT, SP3, RPS15, NOTCH3, JMJD6, ENO1, and DHX30 as the top 10 most significant target genes of the longitudinally upregulated miR-484, miR-107-3p, miR-206, miR-184, miR-1260b, miR-6132 (Figure 7B), with miRNA-target interactions network depicting the complex interaction between the miRNAs and their most significant target genes (Figure 7C). To identify the biological functions of the longitudinally upregulated miRNAs, we performed a KEGG pathway analysis. The results revealed several main groups of KEGG pathways: spliceosome, microRNAs in cancer, glycolysis/gluconeogenesis, central carbon metabolism in cancer, biosynthesis of amino acids, carbon metabolism, renal cell carcinoma, cell cycle, EGFR tyrosine kinase inhibitor resistance, JAK-STAT pathway, as well as fluid shear stress and atherosclerosis, focal adhesion, and viral life cycle—HIV-1 (Figure 7D). It was noteworthy that in this subgroup (VEH/SIV/cART), none of the EC-associated miRNAs were longitudinally altered. 

In the THC/SIV/cART group, 10 EV-associated miRNAs (miR-382-5p, miR-128a-3p, miR-342-3p, miR-100-5p, miR-25, miR-181b-5p, miR-335-5p, miR-128b-3p, miR-20a-5p, miR-210-3p) were significantly downregulated while 1 (miR-1260b) was significantly upregulated, respectively, at 1 MPI (Figure 7E, left). At 5 MPI, THC/cART treatment resulted in the significant downregulation of 5 EV-associated miRNAs (miR-342-3p, miR-122a-5p, miR-100-5p, miR-181b-5p, miR-106a-5p) (Figure 7E, right). Interestingly, miR-342-3p, miR-100-5p, and miR-181b-5p were longitudinally and significantly downregulated in EVs by THC/ART (Figure 7E, black arrows). The magnitude of the alteration is shown in Table 5. 

KPNA2, TCP1, RPS8, RPL26, RPL15, PWP2, NOP2, NDE1, MTOR, and CUL5 were identified as the top 10 most significant target genes of miR-342-3p, miR-100-5p, and miR-181b-5p per MIENTURNET miRTarbase analysis (Figure 7F). Visualization of the EV-associated miRNA-target interaction networks showed complex connections between the miRNAs and their predicted genes (Figure 7G). KEGG pathway analysis revealed a significant enrichment of genes involved in microRNAs in cancer, longevity regulating pathway, glioma, EGFR tyrosine kinase inhibitor resistance, prostate cancer, endocrine resistance, melanoma, cell cycle, bladder cancer, mitophagy and cysteine and methionine metabolism (Figure 7H). Additionally, Reactome pathway analysis identified the top 10 processes associated with miRNAs to be regulation of TP53 degradation, regulation of TP53 expression and degradation, cellular response to heat stress, regulation of TP53 activity, MET activates RAP1 and RAC1, cellular senescence, apoptosis, programmed cell death, CDC6 association with Origin Recognition Complex (ORC) and inhibition of replication initiation of damaged DNA by RB (Supplementary File S1). The top 10 diseases associated with miR-342-3p, miR-100-5p, and miR-181b-5p according to Disease Ontology include telangiectasis, peripheral vascular disease, autosomal dominant disease, thyroid carcinoma, thyroid cancer, autonomic nervous system neoplasm neuroblastoma, peripheral nervous system neoplasm, hematopoietic system disease, and urinary system cancer, and for WikiPathways these include: human thyroid stimulating hormone (TSH) signaling pathway, integrated breast cancer pathway, breast cancer pathway, factors and pathways affecting insulin-like growth factor (IGF1)-Akt signaling, ATM signaling network in development and disease, signaling pathways in glioblastoma, hematopoietic stem cell regulation by GABP alpha/beta complex, integrated cancer pathway, and miRNAs involved in DNA damage response and the retinoblastoma gene in cancer (Supplementary File S1).

In the THC/SIV/cART group, 13 EC-associated miRNAs (miR-99b-5p, miR-192-5p, miR-574, miR-382-3p, miR-19a-3p, miR-190b, miR-188-5p, miR-152-3p, miR-148a-5p, miR-15b-3p, miR-505-5p, miR-145-3p, and miR-676-3p) were significantly downregulated at 1 MPI (Figure 7I, left), while 11 miRNAs (miR-574, miR-7195-3p, miR-95-3p, miR-342-3p, miR-125b-5p, miR-505-5p, miR-1-3p, miR-1249, miR-676-3p, miR-195-5p, and miR-375) were significantly downregulated at 5 MPI (Figure 7I, right). Interestingly, EC-associated miR-676-3p, miR-574, and miR-505-5p were longitudinally and significantly downregulated by THC/cART. The magnitude of the alteration is shown in Table 6. MIENTURNET miRTarbase identified ZNF280D, SIAH1, RPLP2, RPL11, RMDN3, PRKCI, NDUFB8, MAP7D2, KDM4B, and DDX24 as the top 10 most significant target genes of miR-676-3p, miR-574-3p, and miR-505-5p (Figure 7J). Visualization of the three longitudinally downmodulated EC-associated miRNA-target interaction networks depicted complex connections between the miRNAs and their predicted genes (Figure 7K). KEGG pathway analysis suggested that the target genes of the miRNAs longitudinally downmodulated by THC in cART-treated RMs were associated with: transcriptional misregulation in cancer, prostate cancer, parathyroid hormone synthesis, secretion and action, HIF-1 signaling pathway, selenocompound metabolism, Huntington disease, RNA polymerase, nicotine addiction, ferroptosis, and cholesterol metabolism (Figure 7L).

## 3. Discussion

It is known that circulating exmiRNAs are a significant part of body fluids that contribute to the dynamic host responses that occur under various conditions, including HIV infection and drug use. In this follow-up study, we investigated how cART administered in conjunction with THC to SIV-infected RMs altered the abundance and compartmentalization of exmiRNAs in EVs and ECs of SIV-infected RMs. Although our initial study (Manuscript 1) showed that EVs and ECs carry miRNAs and that ECs were the dominant carriers, in this follow-up study, we used cross-sectional and longitudinal miRNA profiling of paired EVs and ECs released by SIV-infected RMs that were treated with THC, cART, or both THC+cART to directly address the impact of viral infection (SIV), cART, and THC on the abundance and compartmentalization of miRNAs in EVs and ECs. We used paired EVs and ECs (isolated from the same blood plasma) to empirically demonstrate that exmiRNAs in blood plasma are carried by both EVs and ECs, with the majority of the exmiRNA being associated with ECs. These miRNA carriers exhibited a unique pattern of change (abundance and compartmentalization) with respect to treatments with THC or cART. 

Analysis of the miRNA abundance (by count) in each carrier at various time points (Pretreatment, 1 MPI, 5 MPI) and treatments as reported on Table 3 showed that let-7a-5p, let-7c-5p, and miR-26a-5p were the most highly abundant miRNAs in EVs, while miR-27a is the most highly abundant in ECs across time points and treatments. The observation on the enrichment of let-7 family of miRNAs with EVs is significant. For instance, Let-7 miRNAs are highly expressed in both glial progenitor cells and astrocytes, and Let-7 participate in priming progenitors for astrogliogenesis [38]. Let-7 miRNAs are involved in regulating CNS inflammation and neurological outcomes. For example, let-7a-5p and let-7c-5p overexpression has suppressed TNFα expression [39,40], while let-7c-5p improved neurological outcomes in a murine model of traumatic brain injury by suppressing neuroinflammation [41]. Moreover, overexpression of Let-7g was shown to preserve blood brain barrier integrity, reduce proinflammatory cytokine release, and prevent immune cell infiltration into infarcted region, leading to improved behavioral outcomes [42,43,44]. With respect to HIV, Swaminathan et al. showed significant down-regulation of Let-7 family of miRNAs in patients with chronic HIV infection compared to healthy controls [45]. Zhang et al. reported that HIV infection suppressed the let-7i/IL-2 axis leading to cell death [46]. Aside from HIV, Let-7d-5p, let-7a, and let-7c decreased over time in agreement with the progression of liver fibrosis in hepatitis C infected people [47]. Additionally, miR-26a-5p and miR-27a have been implicated in HIV pathogenesis. While miR-26a-5p was down-regulated in patients with cART-resistance compared to cART-responsive patients [48], HIV elite controller and healthy controls had a higher expression of miR-27a [49], which distinguished HIV-infected patients from healthy controls [50].

Similar to cellular miRNAs, circulating exmiRNAs are effective mediators of host responses. However, unlike cellular miRNAs, exmiRNAs can be easily shuttled to proximal and distal sites where they exert their functions. Most research efforts on exmiRNAs have been in the field of cancer, where they have been shown to regulate various oncogenic processes [51,52,53,54,55,56,57,58]. In the context of HIV/SIV infection, although the number of miRNAs associated with EVs was lower compared to those associated with ECs across all treatment groups (Figure 1C), the abundance of exmiRNAs within EVs and ECs were not altered by viral infection, viral suppression with cART, or suppression of inflammation by THC (Figure 1D).

Our findings that exmiRNAs are associated with both EVs and ECs, albeit with some differences, support the assertion that various extracellular particles, including lipoproteins [31,32], EVs [29,33], and membraneless condensates (MCs)/extracellular condensates (ECs) [29], are carriers of miRNAs. By their association with EVs and ECs, exmiRNAs may be protected from degradation by RNAses [59]. Indeed, the physical interaction of exmiRNAs with extracellular particles and possibly with protein complexes may significantly protect exmiRNAs from extracellular nucleases and degradation. As shown in this study, both EVs and ECs are associated with proteinaceous molecules, with ECs having significantly higher protein concentrations within and across sample groups (Figure S1E).

It is noteworthy that EVs are the most studied exRNA carriers because EVs are easier to obtain than other extracellular particles, albeit with impurities due to co-purification with non-EVs. Biological functions may have been assigned to EV-associated miRNAs in certain circumstances, but our findings in this study suggest that some of the observed biological effects assigned to EV-associated miRNAs may have possibly been the result of miRNA-associated with EVs (Figure 1, Figure 2, Figure 3, Figure 4, Figure 5, Figure 6 and Figure 7) as well as other exRNA carriers (lipoproteins and MCs/ECs) [29,34]. Therefore, rigorous analysis and cautious interpretation of exmiRNA data, which should consider the isolation method used to obtain the exmiRNA carrier, is critical. In our study, we demonstrated that PPLC (which utilizes gradient beads in a column attached to fraction collector) [29] separated the exmiRNA carriers (EVs and ECs), and that both carriers contain some common and unique miRNAs.

Although exmiRNAs may serve as biomarkers of certain diseases, the source of these miRNAs is unclear. It is also possible that miRNA-associated with non-EV protein complexes, such as ECs, may be released into circulation by lysed or necrotic cells, and such EC-associated miRNAs may have a defined but yet to be discovered role in disease and health. Our study supports the idea that specific miRNAs may be released from cells in response to various pathologies or stimuli, indicating a potentially controlled, active, and specific miRNA release process. In this study, the stimuli tested were SIV, cART, and THC, and all influenced the release of EVs and ECs containing various miRNAs, whose origin remains to be identified. 

The association of specific miRNA with EVs and/or ECs suggests the possible selectivity of exmiRNA interaction with their carriers, although the details are yet to be unraveled. This hypothesis is supported by the findings that the number of exmiRNAs that were associated with ECs is greater than those associated with EVs across groups as follows: ECs>EVs 85.7%, 58.4%, 48.5%, 51.8%, 69.0% for VEH/SIV, SIV/ART, THC/SIV, THC/SIV/ART, and THC/no SIV, respectively (Figure 1C). In addition to the differences in miRNA counts (Table 3), distinct miRNAs were associated with the different carriers—EVs and ECs in different treatment groups, and the levels of these miRNAs also differ. For example, in the VEH/SIV group at 1 MPI, miR-6132, miR-184, miR-542-3p, miR-875-5p, and miR-186-3p were specifically associated with EVs, while miR-223, miR-24-3p, miR-6529-5p, miR-382-5p, miR-154-5p, miR-484, miR-361-5p, miR-222-3p, miR-377-3p, and miR-18b were associated with ECs at 1 MPI. The levels of these miRNA were altered by cART (Figure 3A,B) and THC (Figure 4A,B), suggesting that these agents may dysregulate the host miRNAome. 

Of course, some exmiRNAs are shown to be dysregulated in a variety of malignancies (cancers, diabetes, viral infections) and in a variety of tissues. In this study, the levels of specific miRNAs associated with each carrier provide some degree of insight into HIV/SIV pathogenesis and/or potential mechanisms of disease control. Thus, an improved understanding of which miRNAs are associated with EVs or ECs, and the uptake of these miRNA carriers by different cell types may facilitate the design of studies to assess their roles in diagnostics and therapeutics. Additional studies are therefore needed to evaluate the sources of the exmiRNAs and their functions with respect to HIV/SIV infection. Although bioinformatic target prediction was performed in the current study, in the future we will assess the effect of these plasma derived EVs and ECs on CD4 T cell and monocyte activation in the blood, as these EVs and ECs may come into direct contact with these cells in vivo. 

Conclusion and Translational Relevance

**Figure 8:**
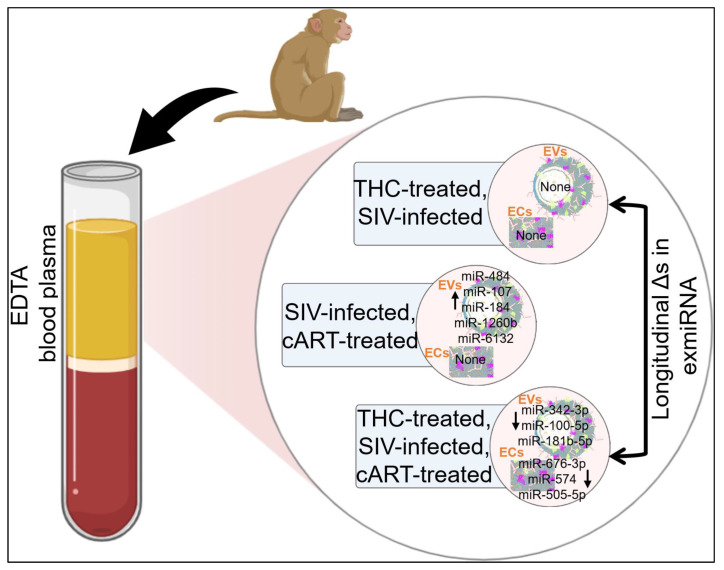
Circulating blood plasma miRNAs and their association with EVs and ECs in SIV-infected rhesus macaques under various treatments—THC, ART, THC, and ART. Parts of this illustration was created with BioRender.com.

This is follow up study, we provided a comprehensive cross-sectional and longitudinal analysis of the host exmiRNA responses to SIV infection and the impact of THC, cART, or THC and cART together on the miRNAome during SIV infection. Clinically, our longitudinal analyses of changes in miRNAs associated with EVs and ECs (Figure 8) identified key sequential miRNA features of SIV infection that may distinguish disease trajectories during viral suppression by cART or inflammation suppression by THC (Table 4, Table 5 and Table 6). Indeed, we found that exmiRNA signatures became longitudinally dysregulated upon SIV infection (Figure 5, Manuscript 1) or during treatment with cART (Figure 7A–D) or THC (7E–L), giving credence to the idea that exmiRNA may contribute to SIV pathogenesis. Indeed, the longitudinal analysis performed in this study provides insight into the pattern of miRNA alterations in EVs and ECs, and revealed potential cause-and-effect relationships in SIV infection and treatment. Furthermore, the consistency of exmiRNA changes from 1 MPI to 5 MPI suggests that these exmiRNAs may serve as biomarkers of HIV/SIV infection and response to treatment with cART and/or THC. Hence, the groups of miRNAs that were longitudinally altered as shown in Table 4, Table 5 and Table 6 will be assessed in future studies for their use as diagnostic or prognostic biomarkers because combining different miRNAs may help to improve biomarker value. It remains to be identified what the biological implications of increasing or decreasing exmiRNA levels in the different carriers are—EVs and ECs following HIV/SIV infection in the presence of cART and/or THC? Nonetheless, the intercellular transfer of exmiRNAs is expected to ensure that miRNA-mediated gene regulation may happen in the absence of miRNA production by the target cells in various contexts.

## 4. Materials and Methods

In this two-part manuscript of the same series, the Materials and Methods used for the follow up study presented in this Manuscript 1 are similar to that of the primary study presented in manuscript 1. Minor differences (if present) are detailed in the various Materials and Methods subsections.

### 4.1. Macaques and Viruses (used for this Study and the Primary Study Presented in Manuscript 1)

Pre-infection and pre-treatment blood samples were collected from a total of 15 age- and weight-matched Mamu-A0∗1^-^/B08^-^/B17^-^ specific-pathogen-free (free of SIV, D retrovirus, STLV and Herpes B) male Indian rhesus macaques (Table 1 of Manuscript 1). The animals were then randomly assigned to five experimental groups. Rhesus macaques in Groups 1 to 4 were infected intravenously with 100 TCID_50_ dose of the CCR5 tropic SIVmac251 (TNPRC virus isolation and production core) (Table 1 of Manuscript 2). Group 1: (JD66, IN24, JH47) and 3 (LM56, LA88, LN60) received twice daily injections of vehicle (VEH/SIV) (1:1:18 of emulphor: alcohol: saline). Groups 2 (JI45, JC85, JT80) and 4 (LA55, KV50, LM85) received twice-daily injections of Δ^9^-THC (THC/SIV), beginning 4 weeks prior to SIV infection until 6 months post-SIV infection [60]. Group 5 (HI78, HN79, HN39) macaques received twice daily injections of THC similar to Groups 2 and 4, but remained SIV-uninfected and served as THC-only controls (Table 1 of Manuscript 2). THC (NIDA/NIH) was prepared as an emulsion using alcohol, emulphor, and saline (1:1:18) as vehicle before use. Chronic administration of VEH (Group 1 & 3) or Δ^9^-THC (Group 2, 4 and 5) was initiated 4 weeks before SIV infection at 0.18mg/kg as used in previous studies [21,60,61]. This dose of Δ^9^-THC was found to eliminate responses in a complex operant behavioral task in almost all of the animals [21,22]. Beginning the day of SIV infection, the THC dose was increased for each subject to 0.32 mg/kg over a period of approximately two weeks when responding was no longer affected by 0.18 mg/kg on a daily basis (i.e., tolerance developed), and it was maintained for the duration of the study. The optimization of the THC dosing in RMs accounts for the development of tolerance during the initial period of administration. In previously published studies [21,22] this dose of THC showed protection, and so the same dose was used in this study. Rhesus macaques in Groups 3 (VEH/SIV/cART) and 4 (THC/SIV/cART) began combination anti-retroviral (cART) treatments (PMPA 20mg/kg, FTC or Emtricitabine 30 mg/kg and Dolutegravir 2.5 mg/kg) at 2 weeks post-SIV infection daily by subcutaneous route until 6 MPI. We want to mention that macaques in Groups 3 and 4 received two injections of (50 mg/kg of anti-alpha4beta7) (LN60 and LM85) or control IgG (LM56) beginning 4 MPI at three-week intervals before the plasma sample at 5 MPI was collected. Nevertheless, we did not see any differences in EV characteristics. Moreover, viral replication was significantly suppressed by cART at this stage. Therefore, we do not expect it to have any effects on EV composition, and accordingly did not see any effects. Macaques LA88, LA55, and KV50 received cART injections until 8 MPI. However, the blood samples used in our study were collected at 5 MPI. 

For studies in Manuscript 1, pre-infection and pre-treatment blood samples as well as 1 MPI and 5 MPI blood samples collected from macaques in Group 1 were used (Table 1 of Manuscript 1).

For studies in Manuscript 2, 1 MPI and 5 MPI blood samples collected from macaques in Groups 1 to 5 were used (Table 1 of Manuscript 2).

### 4.2. Isolation of EVs and ECs (used for this Study and the Primary Study in Manuscript 1)

The EVs and ECs were isolated from EDTA blood plasma samples using the PPLC-based size exclusion chromatography (SEC) as previously described [62]. Briefly, samples were liquefied at room temperature for 30 min and centrifuged at 2, 000× *g* for 10 min and 10,000× *g* for 30 min to remove cellular debris and large vesicles. EVs and ECs were purified using a gravity-packed 7-bead gradient (G-10, G-15, G-25, G-50, G-75, G-100, 2% BCL agarose bead standard) into a 100 cm × 1 cm Econo-Column. Elution was achieved by gravity using 0.1X Phosphate Buffered Saline (PBS, Corning, NY, USA). A fraction of 250 µL were collected, and elution profiles were determined by absorbance measurements at 280 nm. The first peak, which contained EVs, and the last peak, which contained ECs, were independently collected and stored in aliquots at −80 °C until further analysis. 

### 4.3. Nanoparticle Tracking Analysis (NTA) (used for this Study and the Primary Study in Manuscript 1)

EV size, concentration, and zeta-potential (ζ-potential) were measured using ZetaView PMX 110 (Particle Metrix, Mebane, NC, USA) and the corresponding software ZetaView v8.04.02, as previously described [30]. Briefly, the system was calibrated and aligned with 102 nm polystyrene standard beads before the experiment. EV samples were left at room temperature for 30 min to acclimatize before measurement. Samples were diluted to appropriate concentration (1:20,000 to 1:320,000) in 0.1X PBS to reach particle numbers ideal for NTA. All samples were analyzed under the same conditions (room temperature −20 °C to 25 °C), pH 5.8, sensitivity 92, shutter speed 70, and frame rate 30 fps). Triplicate measurements were taken for size and concentration, and each replicate included 11 positions with two cycles of reading at each position. For ζ-potential measurement, data were acquired at least in quintuplicate, and each replicate corresponds to two cycles of reading. EC size, concentration and zeta-potential were not measured as the average EC particle size was lower than the 20 nm detection limit of the Zetaview PMX 110 with the standard laser configuration, therefore preventing the acquisition of accurate and reproducible data (https://www.excilone.com/client/document/particle-metrix--zetaview-brochure-0319_en_540.pdf, accessed on 20 November 2022).

### 4.4. Total RNA Isolation(used for this Study and the Primary Study in Manuscript 1)

Total RNA were isolated from 100 µL of EV and EC samples from each study subject using miRNeasy plasma kit (Qiagen), with the optional on-column DNase-I digestion step. RNA was eluted in 25 µL RNase-free water once andre-eluted with 25 µL RNase-free water. RNA quality control was assessed by Nanodrop 1000 prior to sequencing.

### 4.5. Library Preparation and sRNA Sequencing (used for this Study and the Primary Study in Manuscript 1)

Library preparation and sRNA sequencing were performed as previously described [30] for each subject. Briefly, libraries were amplified by 20 cycles of PCR. Libraries were sequenced in one NextSeq 550 run with the NextSeq 500/550 High Output Kit v2.5 (75 cycles), and sequencing was performed with Single End 75 nt reads and dual 6 nt indexes. Libraries were loaded at 1.5 pM and sequenced with a RealSeq Biosciences (Santa Cruz, CA) custom sequencing primer for read one, and 5% PhiX control was used. FastQ files were trimmed of adapter sequences by using Cutadapt [63] with the following parameters: cutadapt -u 1 -a TGGAATTCTCGGGTGCCAAGG -m 15.

### 4.6. Identification of Common miRNAs (used for this Study and the Primary Study in Manuscript 1)

InteractiVenn web tool [64] was utilized to identify common miRNAs for EVs and ECs between EVs and ECs within each treatment group. Briefly, for a miRNA to be included in this analysis, a cutoff of miRNA distribution count of ≥1 was used. miRNAs for each treatment group were inputted into InteractiVenn to identify common miRNAs by extracellular fraction. 

### 4.7. PCA Plot and Heatmap Generation (used for this Study and the Primary Study in Manuscript 1)

ClustVis web tool [65] was utilized to generate both PCA plots and heatmaps using the same input data. Briefly, log-10 normalized miRNA read counts were inputted into ClustVis for each EVs and ECs and default data pre-processing options were applied. For PCA plot generation: PC1 was assigned to the X-axis and PC2 to the Y-axis, with a margin ration of 0.05. For heatmap generation: rows are centered, and unit variance scaling is applied to rows. Imputation is used for a missing value estimation. Both rows and columns are clustered using correlation distance and average linkage.

### 4.8. Identification of Differentially Enriched miRNAs (used for this Study and the Primary Study in Manuscript 1)

Identification of differentially enriched miRNA was performed as previously described [30] using a cutoff for the sum of reads defined as equal or larger than the number of samples being compared per group times two. Thus, for an miRNA to be included in the analysis, the sum of reads should be ≥12. Subsequently, the read counts were log-10 normalized and two-way ANOVA comparisons between different groups performed in Prism software. The false discovery rate (FDR) was controlled using the method of Benjamini and Hochberg, and was set to <0.05. 

### 4.9. miRNA-Target Enrichment Analysis (used for this Study and the Primary Study in Manuscript 1)

MIENTURNET web tool [66] was utilized for all miRNA-target enrichment analyses. Briefly, miRTarBase was used for miRNA-target enrichment analyses, with a threshold for minimum number of miRNA-target interactions of 2, and a threshold for adjusted FDR of 1. Network analyses were performed with the same filter settings, with the selection of strong evidence only. miRTarBase was again used for subsequent functional enrichment analyses (KEGG, REACTOME, WikiPathways, and Disease Ontology). 

### 4.10. Statistical Analyses (used for this Study and the Primary Study in Manuscript 1)

Statistical tests were performed using the GraphPad Prism (Version 9.3.1) software and are detailed in the figure legends. Sequencing data are provided as Supplementary Files S1–S5. For two-group comparison, unpaired t test with Welch’s correction was used to determine the differences between the groups. Ordinary one-way ANOVA (Brown-Forsythe and Bartlett tests, with Sidak’s multiple comparison tests) was used to determine the differences between multiple groups. 

## Figures and Tables

**Table 1:** Animal IDs, SIV inoculum, infection duration, Δ^9^-THC administration, and ART treatment.

Animal ID	Pre-Infection Sample Used	SIV Inoculum	Group 1 VEH/SIV	Group 2 THC/SIV	Group 3 VEH/SIV/cART	Group 4 THC/SIV /cART	Group 5 THC/no SIV	Post-Infection Sample Collection
JD66	NA	SIVmac251	Yes					1 & 5 MPI
IN24	NA	SIVmac251	Yes					1 & 5 MPI
JH47	NA	SIVmac251	Yes					1 & 5 MPI
JI45	NA	SIVmac251		Yes				1 & 5 MPI
JC85	NA	SIVmac251		Yes				1 & 5 MPI
JT80	NA	SIVmac251		Yes				1 & 5 MPI
LM56	NA	SIVmac251			Yes			1 & 5 MPI
LA88	NA	SIVmac251			Yes			1 & 5 MPI
LN60	NA	SIVmac251			Yes			1 & 5 MPI
LA55	NA	SIVmac251				Yes		1 & 5 MPI
KV50	NA	SIVmac251				Yes		1 & 5 MPI
LM85	NA	SIVmac251				Yes		1 & 5 MPI
HI78	NA	SIVmac251					No	1 & 5 MPT
HN79	NA	SIVmac251					No	1 & 5 MPT
HN39	NA	SIVmac251					No	1 & 5 MPT

NA—Not applicable. MPI—Months post infection. MPT—Months post treatment. Macaques in groups 3 and 4 received two injections of (50 mg/kg of anti-alpha4beta7) (LN60 and LM85) or control IgG (LM56) beginning 4 MPI at three-week intervals before the plasma sample at 5 MPI was collected. Macaques LN60, LM56, and LM85 received ART until 6 MPI. Macaques LA88, LA55, and KV50 received ART until 8 MPI (Table 2). However, samples used for this study were collected at 1 and 5 MPI.

**Table 2:** Animal IDs and additional treatments during the study period.

	Animal IDs	cART Start Timepoint	cART end Timepoint	Control IgG Start Date	Anti-alpha4beta Antibody Start Date
**Cohort 2**					
VEH/SIV/cART	LA88	14 DPI or 0.5 MPI	8 MPI		7 MPI
	LC39	14 DPI or 0.5 MPI	8 MPI		7 MPI
	LD08	14 DPI or 0.5 MPI	8 MPI	7 MPI	
	LE67	14 DPI or 0.5 MPI	8 MPI	7 MPI	
THC/SIV/cART	LA55	14 DPI or 0.5 MPI	8 MPI		7 MPI
	LB61	14 DPI or 0.5 MPI	8 MPI		7 MPI
	LA89	14 DPI or 0.5 MPI	8 MPI	7 MPI	
	KV50	14 DPI or 0.5 MPI	8 MPI	7 MPI	
**Cohort 2**					
VEH/SIV/cART	LM56	14 DPI or 0.5 MPI	6 MPI	4 MPI	
	LH75	14 DPI or 0.5 MPI	6 MPI	4 MPI	
	LN60	14 DPI or 0.5 MPI	6 MPI		4 MPI
	LC48	14 DPI or 0.5 MPI	6 MPI		4 MPI
THC/SIV/cART	LH92	14 DPI or 0.5 MPI	6 MPI	4 MPI	
	LI81	14 DPI or 0.5 MPI	6 MPI	4 MPI	
	LM85	14 DPI or 0.5 MPI	6 MPI		4 MPI
	LJ21	14 DPI or 0.5 MPI	6 MPI		4 MPI

MPI—Months post infection. DPI—Days post infection. Macaques LN60 and LM85 received two injections of 50 mg/kg of anti-alpha4beta7. Macaque LM56 received two injections of 50 mg/kg of control IgG. These injections were given at 4 MPI at three-week intervals before the plasma sample at 5 MPI was collected. Macaques LN60, LM56, and LM85 received ART until 6 MPI. Macaques LA88, LA55 and KV50 received cART until 8 MPI. But samples used for this study were collected at 1 and 5 MPI.

**Table 3:** Most highly enriched miRNA by counts in EVs and ECs in various groups at different times.

Treatment/ Time	miRNA ID	miRNA Counts	miRNA ID	miRNA Counts
**VEH/SIV**	**EVs**		**ECs**	
**Pre**	mml-miR-26a-5p	2496	mml-miR-27a-3p	9578
	mml-let-7a-5p	2368	mml-miR-27b-3p	9435
	mml-let-7c-5p	2360	mml-miR-191-5p	8459
**1 MPI**	mml-miR-26a-5p	1603	mml-miR-16-5p	8264
	mml-let-7c-5p	1588	mml-miR-191-5p	8171
	mml-let-7a-5p	1578	mml-miR-27a-3p	6244
**5 MPI**	mml-let-7c-5p	1483	mml-miR-27a-3p	9669
	mml-let-7a-5p	1476	mml-miR-191-5p	9617
	mml-miR-26a-5p	1329	mml-miR-27b-3p	9531
**VEH/SIV/cART**	**EVs**		**ECs**	
**Pre**	mml-let-7a-5p	402	mml-miR-16-5p	2815
	mml-let-7c-5p	398	mml-miR-27a-3p	1692
	mml-miR-26a-5p	299	mml-miR-27b-3p	1674
**1 MPI**	mml-let-7c-5p	1367	mml-miR-16-5p	3389
	mml-let-7a-5p	1353	mml-miR-451	1962
	mml-miR-26a-5p	651	mml-miR-27a-3p	1689
**5 MPI**	mml-let-7c-5p	560	mml-miR-16-5p	2589
	mml-let-7a-5p	550	mml-miR-451	1710
	mml-miR-26a-5p	290	mml-miR-191-5p	1590
**THC/SIV**	**EVs**		**ECs**	
**Pre**	mml-miR-26a-5p	6930	mml-miR-27a-3p	10775
	mml-let-7a-5p	5818	mml-miR-27b-3p	10656
	mml-let-7c-5p	5796	mml-miR-16-5p	10181
**1 MPI**	mml-let-7a-5p	711	mml-miR-191-5p	5314
	mml-let-7c-5p	709	mml-miR-16-5p	3706
	mml-miR-26a-5p	642	mml-miR-27a-3p	3615
**5 MPI**	mml-let-7c-5p	765	mml-miR-16-5p	2269
	mml-let-7a-5p	761	mml-miR-191-5p	2222
	mml-miR-26a-5p	640	mml-miR-27a-3p	1577
**THC/SIV/cART**	**EVs**		**ECs**	
**Pre**	mml-let-7a-5p	3856	mml-miR-451	18618
	mml-let-7c-5p	3783	mml-miR-16-5p	18182
	mml-miR-26a-5p	1872	mml-miR-191-5p	6051
**1 MPI**	mml-let-7c-5p	1332	mml-miR-16-5p	3031
	mml-let-7a-5p	1321	mml-miR-191-5p	2945
	mml-miR-26a-5p	1073	mml-miR-27a-3p	2291
**5 MPI**	mml-let-7a-5p	1792	mml-miR-16-5p	3912
	mml-let-7c-5p	1789	mml-miR-191-5p	3267
	mml-miR-26a-5p	1648	mml-miR-27a-3p	2376
**THC/no SIV**	**EVs**		**ECs**	
**Pre**	mml-let-7a-5p	2078	mml-miR-27a-3p	14099
	mml-let-7c-5p	2069	mml-miR-27b-3p	13775
	mml-miR-26a-5p	1777	mml-miR-191-5p	13511
**1 MPI**	mml-let-7c-5p	2763	mml-miR-191-5p	20060
	mml-let-7a-5p	2757	mml-miR-16-5p	19156
	mml-miR-26a-5p	2240	mml-miR-27a-3p	18669
**5 MPI**	mml-let-7a-5p	1705	mml-miR-191-5p	27536
	mml-let-7c-5p	1694	mml-miR-16-5p	18294
	mml-miR-26a-5p	1477	mml-miR-27a-3p	17992

**Table 4:** Longitudinal regulation of EV-associated miRNAs by ART in SIV-infected RMs.

	Regulation	FC (log2)	*p* Value	−log (*p* Value)	Citation
**miR-484**					
1 MPI	Up	3.473	0.0001	4.000	This study
5 MPI	Up	2.567	0.011	1.963	This study
**miR-107**					
1 MPI	Up	1.755	0.0056	2.252	This study
5 MPI	Up	1.504	0.014	1.854	This study
**miR-206**					
1 MPI	Up	3.429	0.0001	4.000	This study
5 MPI	Up	2.846	0.0014	2.854	This study
**miR-184**					
1 MPI	Up	1.674	0.001	3.000	This study
5 MPI	Up	2.567	0.0001	4.000	This study
**miR-1260b**					
1 MPI	Up	2.772	0.0086	2.066	This study
5 MPI	Up	2.326	0.0378	1.423	This study
**miR-6132**					
1 MPI	Up	3.668	0.0001	4.000	This study
5 MPI	Up	3.201	0.0001	4.000	This study

**Table 5:** Longitudinal regulation of EV-associated miRNAs by THC in virally suppressed RMs.

	Regulation	FC (log2)	*p* Value	−log (*p* Value)	Citation
**miR-342-3p**					
1 MPI	Down	−3.012	0.0105	1.979	This study
5 MPI	Down	−2.010	0.0444	1.353	This study
**miR-100-5p**					
1 MPI	Down	−1.805	0.0067	2.174	This study
5 MPI	Down	−1.657	0.0175	1.757	This study
**miR-181b-5p**					
1 MPI	Down	−2.266	0.0057	2.244	This study
5 MPI	Down	−1.680	0.028	1.587	This study

**Table 6:** Longitudinal regulation of EC-associated miRNAs by THC in virally suppressed RMs.

	Regulation	FC (log2)	*p* Value	−log (*p* Value)	Citation
**miR-676-3p**					
1 MPI	Down	−1.770	0.0222	1.654	This study
5 MPI	Down	−2.771	0.0175	1.757	This study
**miR-574**					
1 MPI	Down	−2.858	0.0031	2.509	This study
5 MPI	Down	−1.856	0.0323	1.491	This study
**miR-505-5p**					
1 MPI	Down	−2.736	0.0073	2.137	This study
5 MPI	Down	−2.736	0.0209	1.680	This study

## Data Availability

The sRNA-Seq datasets are included within the article and its additional files.

## Supplementary Materials

The following supporting information can be downloaded at: https://www.mdpi.com/article/10.3390/v15030623/s1, Supplemental File S1. Target enrichment analyses for miR-342-3p, miR-100-5p and miR-181b-5p; Supplemental File S2. VEH_SIV EV and EC raw miRNA counts; Supplemental File S3. THC_SIV EV and EC raw miR counts; Supplemental File S4. SIV_ART EV and EC raw miR counts; Supplemental File S5. THC_SIV_ART EV and EC raw miR counts; Supplemental File S6. THC EV and EC raw miRNA counts.

## Abbreviations

ECs	Extracellular condensates
EVs	Extracellular vesicles
ART	Antiretroviral therapy
THC	Delta-9-tetrahydrocannabinol
VEH	Vehicle
